# Evaluation of Polygenic Risk Scores for Breast and Ovarian Cancer Risk Prediction in *BRCA1* and *BRCA2* Mutation Carriers

**DOI:** 10.1093/jnci/djw302

**Published:** 2017-03-09

**Authors:** Karoline B. Kuchenbaecker, Lesley McGuffog, Daniel Barrowdale, Andrew Lee, Penny Soucy, Sue Healey, Joe Dennis, Michael Lush, Mark Robson, Amanda B. Spurdle, Susan J. Ramus, Nasim Mavaddat, Mary Beth Terry, Susan L. Neuhausen, Ute Hamann, Melissa Southey, Esther M. John, Wendy K. Chung, Mary B. Daly, Saundra S. Buys, David E. Goldgar, Cecilia M. Dorfling, Elizabeth J. van Rensburg, Yuan Chun Ding, Bent Ejlertsen, Anne-Marie Gerdes, Thomas V. O. Hansen, Susan Slager, Emily Hallberg, Javier Benitez, Ana Osorio, Nancy Cohen, William Lawler, Jeffrey N. Weitzel, Paolo Peterlongo, Valeria Pensotti, Riccardo Dolcetti, Monica Barile, Bernardo Bonanni, Jacopo Azzollini, Siranoush Manoukian, Bernard Peissel, Paolo Radice, Antonella Savarese, Laura Papi, Giuseppe Giannini, Florentia Fostira, Irene Konstantopoulou, Julian Adlard, Carole Brewer, Jackie Cook, Rosemarie Davidson, Diana Eccles, Ros Eeles, Steve Ellis, EMBRACE, Debra Frost, Shirley Hodgson, Louise Izatt, Fiona Lalloo, Kai-ren Ong, Andrew K. Godwin, Norbert Arnold, Bernd Dworniczak, Christoph Engel, Andrea Gehrig, Eric Hahnen, Jan Hauke, Karin Kast, Alfons Meindl, Dieter Niederacher, Rita Katharina Schmutzler, Raymonda Varon-Mateeva, Shan Wang-Gohrke, Barbara Wappenschmidt, Laure Barjhoux, Marie-Agnès Collonge-Rame, Camille Elan, GEMO Study Collaborators, Lisa Golmard, Emmanuelle Barouk-Simonet, Fabienne Lesueur, Sylvie Mazoyer, Joanna Sokolowska, Dominique Stoppa-Lyonnet, Claudine Isaacs, Kathleen B. M. Claes, Bruce Poppe, Miguel de la Hoya, Vanesa Garcia-Barberan, Kristiina Aittomäki, Heli Nevanlinna, Margreet G. E. M. Ausems, J. L. de Lange, Encarna B. Gómez Garcia, HEBON, Frans B. L. Hogervorst, Carolien M. Kets, Hanne E. J. Meijers-Heijboer, Jan C. Oosterwijk, Matti A. Rookus, Christi J. van Asperen, Ans M. W. van den Ouweland, Helena C. van Doorn, Theo A. M. van Os, Ava Kwong, Edith Olah, Orland Diez, Joan Brunet, Conxi Lazaro, Alex Teulé, Jacek Gronwald, Anna Jakubowska, Katarzyna Kaczmarek, Jan Lubinski, Grzegorz Sukiennicki, Rosa B. Barkardottir, Jocelyne Chiquette, Simona Agata, Marco Montagna, Manuel R. Teixeira, KConFab Investigators, Sue Kyung Park, Curtis Olswold, Marc Tischkowitz, Lenka Foretova, Pragna Gaddam, Joseph Vijai, Georg Pfeiler, Christine Rappaport-Fuerhauser, Christian F. Singer, Muy-Kheng M. Tea, Mark H. Greene, Jennifer T. Loud, Gad Rennert, Evgeny N. Imyanitov, Peter J. Hulick, John L. Hays, Marion Piedmonte, Gustavo C. Rodriguez, Julie Martyn, Gord Glendon, Anna Marie Mulligan, Irene L. Andrulis, Amanda Ewart Toland, Uffe Birk Jensen, Torben A. Kruse, Inge Sokilde Pedersen, Mads Thomassen, Maria A. Caligo, Soo-Hwang Teo, Raanan Berger, Eitan Friedman, Yael Laitman, Brita Arver, Ake Borg, Hans Ehrencrona, Johanna Rantala, Olufunmilayo I. Olopade, Patricia A. Ganz, Robert L. Nussbaum, Angela R. Bradbury, Susan M. Domchek, Katherine L. Nathanson, Banu K. Arun, Paul James, Beth Y. Karlan, Jenny Lester, Jacques Simard, Paul D. P. Pharoah, Kenneth Offit, Fergus J. Couch, Georgia Chenevix-Trench, Douglas F. Easton, Antonis C. Antoniou

**Affiliations:** **Affiliations of authors:** The Wellcome Trust Sanger Institute, Wellcome Trust Genome Campus, Hinxton, Cambridge, UK (KBK); Department of Public Health and Primary Care, University of Cambridge, Cambridge, UK (KBK, LM, DB, AL, JD, ML, NM, DFE, ACA); Genomics Center, Centre Hospitalier Universitaire de Québec Research Center and Laval University, Quebec City, Quebec, Canada (PS, JSi); Department of Genetics, QIMR Berghofer Medical Research Institute, Brisbane, Australia (SHe); Clinical Genetics, Service, Department of Medicine, Memorial Sloan Kettering Cancer Center, New York, NY (MR); Department of Genetics and Computational Biology, QIMR Berghofer Medical Research Institute, Brisbane, Australia (ABS, GCT); Department of Preventive Medicine, Keck School of Medicine, University of Southern California, Los Angeles, CA (SJR); Department of Epidemiology, Columbia University, New York, NY (MBT); Department of Population Sciences, Beckman Research Institute of City of Hope, Duarte, CA (SLN, YCD); Molecular Genetics of Breast Cancer, German Cancer Research Center, Heidelberg, Germany (UH); Genetic Epidemiology Laboratory, Department of Pathology, University of Melbourne, Parkville, VIC, Australia (MS); Department of Epidemiology, Cancer Prevention Institute of California, Fremont, CA (EMJ); Departments of Pediatrics and Medicine, Columbia University, New York, NY (WKC); Department of Clinical Genetics, Fox Chase Cancer Center, Philadelphia, PA (MBD); Department of Medicine, Huntsman Cancer Institute, Salt Lake City, UT (SSB); Department of Dermatology, University of Utah School of Medicine, Salt Lake City, UT (DEG); Cancer Genetics Laboratory, Department of Genetics, University of Pretoria, Arcadia, South Africa (CMD); Cancer Genetics Laboratory, Department of Genetics, University of Pretoria, Arcadia, South Africa (EJvR); Department of Oncology (BE), Department of Clincial Genetics (AMG), and Center for Genomic Medicine (TVOH), Rigshospitalet, Copenhagen University Hospital, Copenhagen, Denmark; Department of Health Sciences Research, Mayo Clinic, Rochester, MN (SS, EHal, CO); Human Genetics Group (JBe, AO) and Human Genotyping Unit, Human Cancer Genetics Program (JBe), Spanish National Cancer Centre , Madrid, Spain; Biomedical Network on Rare Diseases, CIBERER, Madrid, Spain (JBe, AO); City of Hope Clinical Cancer Genomics Community Research Network, Duarte, CA (NC, WL); Clinical Cancer Genetics, City of Hope, Duarte, California (JNW); IFOM, Institute of Molecular Oncology, Italian Foundation for Cancer Research, Milan, Italy (PP, VP); Cogentech Cancer Genetic Test Laboratory, Milan, Italy (VP); Centro di Riferimento Oncologico, IRCCS, Aviano, Italy (RDo); University of Queensland Diamantina Institute, Translational Research Institute, Brisbane, Australia (RDo); Division of Cancer Prevention and Genetics, Istituto Europeo di Oncologia, Milan, Italy (MB, BB); Unit of Medical Genetics (JAz, SMan, BPe) and Unit of Molecular Bases of Genetic Risk and Genetic Testing (PR), Department of Preventive and Predictive Medicine, Fondazione Istituto di Ricovero e Cura a Carattere Scientifico, Istituto Nazionale Tumori, Milan, Italy;Unit of Genetic Counselling, Medical Oncology Department, Istituto Nazionale Tumori Regina Elena, Rome, Italy (AS); Unit of Medical Genetics, Department of Biomedical, Experimental and Clinical Sciences, University of Florence, Florence, Italy (LP); Department of Molecular Medicine, University La Sapienza, Rome, Italy (GGi); Molecular Diagnostics Laboratory, Institute of Nuclear and Radiological Sciences and Technology, National Centre for Scientific Research Demokritos, Aghia Paraskevi Attikis, Athens, Greece (FF, IK); Yorkshire Regional Genetics Service, Chapel Allerton Hospital, Leeds, UK (JAd); Department of Clinical Genetics, Royal Devon and Exeter Hospital, Exeter, UK (CB); Sheffield Clinical Genetics Service, Sheffield Children’s Hospital, Sheffield, UK (JCo); Department of Clinical Genetics, South Glasgow University Hospitals, Glasgow, UK (RDa); University of Southampton Faculty of Medicine, Southampton University Hospitals NHS Trust, Southampton, UK (DE); Oncogenetics Team, The Institute of Cancer Research and Royal Marsden NHS Foundation Trust, London UK (RE); Centre for Cancer Genetic Epidemiology, Department of Public Health and Primary Care, University of Cambridge, Strangeways Research Laboratory, Cambridge, UK (SE, EMBRACE, DF); Medical Genetics Unit, St George's, University of London, London, UK (SHo); Clinical Genetics, Guy’s and St. Thomas’ NHS Foundation Trust, London, UK (LI); Genetic Medicine, Manchester Academic Health Sciences Centre, Central Manchester University Hospitals NHS Foundation Trust, Manchester, UK (FLa); West Midlands Regional Genetics Service, Birmingham Women’s Hospital Healthcare NHS Trust, Edgbaston, Birmingham, UK (KrO); Department of Pathology and Laboratory Medicine, University of Kansas Medical Center, Kansas City, KS (AKG); Department of Gynaecology and Obstetrics, University Hospital of Schleswig-Holstein, Campus Kiel, Christian-Albrechts University Kiel, Germany (NA); Institute of Human Genetics, University of Münster, Münster, Germany (BD); Institute for Medical Informatics, Statistics and Epidemiology, University of Leipzig, Germany (CEn); Centre of Familial Breast and Ovarian Cancer, Department of Medical Genetics, Institute of Human Genetics, University Würzburg, Germany (AG); Center of Familial Breast and Ovarian Cancer, Centre for Integrated Oncology, Center for Molecular Medicine Cologne, University Hospital Cologne, Medical Faculty, Cologne, Germany (EHah, JH, RKS, BW); Department of Gynaecology and Obstetrics, University Hospital Carl Gustav Carus, Technical University Dresden, Germany (KKas); Department of Gynaecology and Obstetrics, Division of Tumor Genetics, Klinikum Rechts der Isar, Technical University Munich, Germany (AM); Department of Gynaecology and Obstetrics, University Hospital Düsseldorf, Heinrich-Heine University Düsseldorf, Germany (DN); Institute of Human Genetics, Campus Virchov Klinikum, Charite Berlin, Germany (RVM); Department of Gynaecology and Obstetrics, University Hospital Ulm, Germany (SWG); Bâtiment Cheney D, Centre Léon Bérard, Lyon, France (LB); Lyon Neuroscience Research Center- CRNL, Inserm U1028, CNRS UMR5292, University of Lyon, Lyon, France (SMaz); Service de Génétique Biologique, CHU de Besançon, Besançon, France (ACR); Service de Génétique, Institut Curie, Paris, France (CEl, LG); Institut Curie, Department of Tumour Biology, Paris, France (GEMO, DSL); Institut Curie, INSERM U830, Paris, France (GEMO, DSL); Université Paris Descartes, Sorbonne Paris Cité, France (GEMO, DSL); Oncogénétique, Institut Bergonié, Bordeaux, France (EBS); Genetic Epidemiology of Cancer team, Inserm U900, Paris, France (FLe); Institut Curie, Paris, France (FLe); Mines ParisTech, Fontainebleau, France (FLe); Laboratoire de Génétique Médicale, Nancy Université, Centre Hospitalier Régional et Universitaire, Vandoeuvre-les-Nancy, France (JSo); Lombardi Comprehensive Cancer Center, Georgetown University, Washington, DC (CI); Center for Medical Genetics, Ghent University, Gent, Belgium (KBMC, BPo); Molecular Oncology Laboratory, Hospital Clinico San Carlos, El Instituto de Investigación Sanitaria del Hospital Clínico San Carlos, Madrid, Spain (MdlH, VGB); Department of Clinical Genetics, Helsinki University Hospital, HUS, Finland (KA); Department of Obstetrics and Gynecology, University of Helsinki and Helsinki University Hospital, Biomedicum Helsinki, HUS, Finland (HN); Department of Medical Genetics, University Medical Center Utrecht, Utrecht, the Netherlands (MGEMA); Department of Epidemiology (JLdL) and Coordinating Center, Hereditary Breast and Ovarian Cancer Research Group Netherlands (HEBON), Netherlands Cancer Institute, Amsterdam, the Netherlands (JLdL); Department of Clinical Genetics and GROW, School for Oncology and Developmental Biology, MUMC, Maastricht, the Netherlands (EBGG); Family Cancer Clinic, Netherlands Cancer Institute, Amsterdam, the Netherlands (FBLH); Department of Human Genetics, Radboud University Nijmegen Medical Centre, Nijmegen, the Netherlands (CMK); Department of Clinical Genetics, VU University Medical Centre, Amsterdam, the Netherlands (HEJMH); Department of Genetics, University Medical Center, Groningen University, Groningen, the Netherlands (JCO); Department of Epidemiology, Netherlands Cancer Institute, Amsterdam, the Netherlands (MAR); Department of Clinical Genetics Leiden University Medical Center Leiden, Leiden, the Netherlands (CJvA); Department of Clinical Genetics, Family Cancer Clinic, Erasmus University Medical Center, Rotterdam, the Netherlands (AMWvdO); Department of Gynaecology, Family Cancer Clinic, Erasmus MC Cancer Institute, Rotterdam, the Netherlands (HCvD); Department of Clinical Genetics, Academic Medical Center, Amsterdam, the Netherlands (TAMvO); Hong Kong Hereditary Breast Cancer Family Registry, Hong Kong (AK); Cancer Genetics Center, Hong Kong Sanatorium and Hospital, Hong Kong (AK); Department of Surgery, The University of Hong Kong, Hong Kong (AK); Department of Molecular Genetics, National Institute of Oncology, Budapest, Hungary (EO); Oncogenetics Group, Vall d’Hebron Institute of Oncology (VHIO), Universitat Autònoma de Barcelona, Vall d’Hebron University Hospital, Barcelona, Spain (OD); Genetic Counseling Unit, Hereditary Cancer Program, Institut d'Investigació Biomèdica de Girona, Catalan Institute of Oncology, Girona, Spain (JBr); Molecular Diagnostic Unit (CL) and Genetic Counseling Unit (AT), Hereditary Cancer Program, Bellvitge Biomedical Research Institute, Catalan Institute of Oncology, L'Hospitalet, Barcelona, Spain; Department of Genetics and Pathology, Pomeranian Medical University, Szczecin, Poland (JG, AJ, KKac, JLu, GS); Laboratory of Cell Biology, Department of Pathology, Reykjavik, Iceland (RBB); Biomedical Centre, Faculty of Medicine, University of Iceland, Reykjavik, Iceland (RBB); Unité de Recherche en Santé des Populations, Centre des Maladies du Sein Deschênes-Fabia, Hôpital du Saint-Sacrement, Québec, Canada (JCh); Immunology and Molecular Oncology Unit, Veneto Institute of Oncology IOV - IRCCS, Padua, Italy (SA, MM); Department of Genetics, Portuguese Oncology Institute of Porto, Porto, Portugal (MRT); Biomedical Sciences Institute (ICBAS), University of Porto, Porto, Portugal (MRT); Kathleen Cuningham Consortium for Research into Familial Breast Cancer, Peter MacCallum Cancer Center, Melbourne, Australia (KConFab); Department of Preventive Medicine, Department of Biomedical Science, and Cancer Research Institute, Seoul National University, Seoul, Korea (SKP); Program in Cancer Genetics, Departments of Human Genetics and Oncology, McGill University, Montreal, Quebec, Canada (MTi); Department of Cancer Epidemiology and Genetics, Masaryk Memorial Cancer Institute, Brno, Czech Republic (LF); Clinical Cancer Genetics Laboratory, Memorial Sloan Kettering Cancer Center, New York, NY (PG); Clinical Genetics Research Laboratory, Department of Medicine, Memorial Sloan Kettering Cancer Center, New York, NY (JV); Department of Gynecology and Gynecological Oncology, Comprehensive Cancer Center (GP), and Department of OB/GYN (CRF, CFS, MKMT), Medical University of Vienna, Vienna, Austria; Clinical Genetics Branch, Division of Cancer Epidemiology and Genetics, National Cancer Institute, National Institutes of Health, Rockville, MD (MHG, JTL); Clalit National Israeli Cancer Control Center and Department of Community Medicine and Epidemiology, Carmel Medical Center and B. Rappaport Faculty of Medicine, Haifa, Israel (GR); N. N. Petrov Institute of Oncology, St. Petersburg, Russia (ENI); Center for Medical Genetics, NorthShore University HealthSystem, University of Chicago Pritzker School of Medicine, Evanston, IL (PJH); The Ohio State University Comprehensive Cancer Center Arthur C. James Cancer Hospital and Richard J. Solove Research Institute Biomedical Research Tower, Columbus, OH (JLH); NRG Oncology, Statistics and Data Management Center, Roswell Park Cancer Institute, Buffalo, NY (MP); Division of Gynecologic Oncology, NorthShore University HealthSystem, University of Chicago, Evanston, IL (GCR); ANZGOG, NHMRC Clinical Trials Centre, Camperdown, Australia (JM); Ontario Cancer Genetics Network, Lunenfeld-Tanenbaum Research Institute, Mount Sinai Hospital, Toronto, Ontario, Canada (GGl); Laboratory Medicine Program, University Health Network, Toronto, Ontario, Canada (AMM); Department of Laboratory Medicine and Pathobiology, University of Toronto, Toronto, ON, Canada (AMM); Lunenfeld-Tanenbaum Research Institute, Mount Sinai Hospital, Toronto, Ontario, Canada (ILA); Departments of Molecular Genetics and Laboratory Medicine and Pathobiology, University of Toronto, Ontario, Canada (ILA); Division of Human Cancer Genetics, Departments of Internal Medicine and Molecular Virology, Immunology and Medical Genetics, Comprehensive Cancer Center, The Ohio State University, Columbus, OH (AET); Department of Clinical Genetics, Aarhus University Hospital, Aarhus N, Denmark (UBJ); Department of Clinical Genetics, Odense University Hospital, Odense C, Denmark (TAK, MTh); Section of Molecular Diagnostics, Clinical Biochemistry, Aalborg University Hospital, Aalborg, Denmark (ISP); Section of Genetic Oncology, Department of Laboratory Medicine, University and University Hospital of Pisa, Pisa Italy (MAC); Cancer Research Initiatives Foundation, Sime Darby Medical Centre, Subang Jaya, Malaysia (SHT); University Malaya Cancer Research Institute, University Malaya, Kuala Lumpur, Malaysia (SHT); The Institute of Oncology, Chaim Sheba Medical Center, Ramat Gan, Israel (RB); The Susanne Levy Gertner Oncogenetics Unit, Institute of Human Genetics, Chaim Sheba Medical Center, Ramat Gan, Israel (EF); Sackler Faculty of Medicine, Tel Aviv University, Ramat Aviv, Israel (EF); The Susanne Levy Gertner Oncogenetics Unit, Institute of Human Genetics, Chaim Sheba Medical Center, Ramat Gan, Israel (YL); Department of Oncology, Karolinska University Hospital, Stockholm, Sweden (BA); Department of Oncology, Clinical Sciences, Lund University and Skåne University Hospital, Lund, Sweden (AB); Department of Clinical Genetics, Lund University Hospital, Lund, Sweden (HE); Department of Clinical Genetics, Karolinska University Hospital L5:03, Stockholm, Sweden (JR); University of Chicago Medical Center, Chicago, IL (OIO); UCLA Schools of Medicine and Public Health, Division of Cancer Prevention and Control Research, Jonsson Comprehensive Cancer Center, Los Angeles, CA (PAG); Medical Sciences, University of California, San Francisco, San Francisco, CA (RLN); Department of Medicine, Abramson Cancer Center, Perelman School of Medicine at the University of Pennsylvania, Philadelphia, PA (ARB, SMD, KLN); Department of Breast Medical Oncology and Clinical Cancer Genetics Program, University Of Texas MD Andersson Cancer Center, Houston, TX (BKA); Familial Cancer Centre, Peter MacCallum Cancer Centre, Melbourne, VIC, Australia (PJ); Women's Cancer Program at the Samuel Oschin Comprehensive Cancer Institute, Cedars-Sinai Medical Center, Los Angeles, CA (BYK, JLe); Department of Oncology, University of Cambridge, Cambridge, UK (PDPP); Clinical Genetics Research Laboratory, Department of Medicine, Cancer Biology and Genetics, Memorial Sloan-Kettering Cancer Center, New York, NY 10044 (KO); Department of Laboratory Medicine and Pathology, and Health Sciences Research, Mayo Clinic, Rochester, MN (FJC)

## Abstract

**Background:** Genome-wide association studies (GWAS) have identified 94 common single-nucleotide polymorphisms (SNPs) associated with breast cancer (BC) risk and 18 associated with ovarian cancer (OC) risk. Several of these are also associated with risk of BC or OC for women who carry a pathogenic mutation in the high-risk BC and OC genes BRCA1 or BRCA2. The combined effects of these variants on BC or OC risk for *BRCA1* and *BRCA2* mutation carriers have not yet been assessed while their clinical management could benefit from improved personalized risk estimates.

**Methods:** We constructed polygenic risk scores (PRS) using BC and OC susceptibility SNPs identified through population-based GWAS: for BC (overall, estrogen receptor [ER]–positive, and ER-negative) and for OC. Using data from 15 252 female *BRCA1* and 8211 *BRCA2* carriers, the association of each PRS with BC or OC risk was evaluated using a weighted cohort approach, with time to diagnosis as the outcome and estimation of the hazard ratios (HRs) per standard deviation increase in the PRS.

**Results:** The PRS for ER-negative BC displayed the strongest association with BC risk in *BRCA1* carriers (HR = 1.27, 95% confidence interval [CI] = 1.23 to 1.31, *P = *8.2×10^−53^). In *BRCA2* carriers, the strongest association with BC risk was seen for the overall BC PRS (HR = 1.22, 95% CI = 1.17 to 1.28, *P = *7.2×10^−20^). The OC PRS was strongly associated with OC risk for both *BRCA1* and *BRCA2* carriers. These translate to differences in absolute risks (more than 10% in each case) between the top and bottom deciles of the PRS distribution; for example, the OC risk was 6% by age 80 years for *BRCA2* carriers at the 10th percentile of the OC PRS compared with 19% risk for those at the 90th percentile of PRS.

**Conclusions:** BC and OC PRS are predictive of cancer risk in *BRCA1* and *BRCA2* carriers. Incorporation of the PRS into risk prediction models has promise to better inform decisions on cancer risk management.

Women who carry a pathogenic mutation in the BRCA1 or BRCA2 gene are at high risk of developing breast and ovarian cancers. The clinical management of healthy women with a *BRCA1* or *BRCA2* mutation involves a combination of frequent screening, risk-reducing surgeries, and chemoprevention (1). Important decisions include whether or not to undergo preventive mastectomy and the age at which to undergo risk-reducing salpingo-oophorectomy (RRSO). These choices are invasive, have substantial side effects, and are associated with adverse psychological effects (2–6). Improved personalized cancer risk estimates may help to identify women at particularly high risk or with high risk of disease at early ages who may benefit from early intervention as well as women at lower risk who may opt to delay surgery or chemoprevention (7). This could be achieved by incorporating risk-modifying factors into risk prediction.

Population-based genome-wide association studies have identified 94 common breast and 18 ovarian cancer susceptibility loci (8–10). While a smaller number of these loci were associated with risk in *BRCA1* and *BRCA2* mutation carriers at stringent statistical significance thresholds, the effect sizes in carriers are generally similar to those in the general population, once differences in the distributions of breast tumor estrogen receptor status in mutation carriers and noncarriers are taken into account (9,11). Individually the identified breast and ovarian cancer risk-modifying variants confer only small to modest increases in risk. However, their effects can be combined into polygenic risk scores (PRSs), which may be associated with much larger relative risks (12,13). Prior to the clinical implementation of these findings, it is important to assess the predictive utility of PRS in terms of discrimination, calibration, and potential for risk stratification (14).

Because women with *BRCA1* and *BRCA2* mutations are already at high risk of developing breast and ovarian cancers, the combined effects of risk-modifying variants could lead to much larger differences in the absolute risk of developing the disease as compared with the general population (12,13,15,16). Earlier studies investigating the effect of PRS on the absolute risks of breast and ovarian cancer risks of *BRCA1* and *BRCA2* mutation carriers demonstrated potential for risk stratification (13,17–19). However, these have been based on small numbers of single-nucleotide polymorphisms (SNPs; <15) and most were restricted to theoretical projections of the PRS association rather than empirical evaluations.

In this study, we developed different PRSs for breast and ovarian cancer as well as estrogen receptor (ER)–specific PRS based on reported susceptibility loci from population-based studies and evaluated their associations with risks for *BRCA1* and *BRCA2* carriers. We estimated absolute risks of developing breast and ovarian cancer for individuals with different values of the PRS in order to assess whether these PRS provide clinically useful risk stratification of mutation carriers.

## Methods

### Study Population

Eligible study subjects included in the Consortium of Investigators of Modifiers of *BRCA1*/2 (CIMBA) are female carriers of a pathogenic mutation in either *BRCA1* or *BRCA2* who are age 18 years or older. Mutation carriers were recruited by 56 study centers in 26 countries. The majority were recruited through cancer genetics clinics, and enrolled into national or regional studies. We used data from 15 252 *BRCA1* (breast cancer = 7797, ovarian cancer = 2462) and 8211 *BRCA2* (breast cancer = 4330, ovarian cancer = 631) mutation carriers who were genotyped with the iCOGS array. Quality control has been described in detail elsewhere (11,13,18). Each of the host institutions recruited mutation carriers under protocols approved by local ethics review boards. Written informed consent was obtained from all subjects. Only samples of European ancestry were included in the present analysis.

### Polygenic Risk Scores

The effects of cancer susceptibility variants on cancer risks for mutation carriers were combined into PRS. The PRS for individual i was defined as the sum of the number of risk alleles across k variants weighted by the effect size of each variant:

$${PRS}_{i}=\beta_{1}g_{1i}+\ldots +\beta_{k}g_{ki},$$

where $g_{li}$ is the genotype of person *i* for variant *l*, expressed as the number of effect alleles (0, 1, or 2), and $\beta_{l}$ is the per-allele log risk ratio (odds ratio [OR] or hazard ratio [HR]) (Supplementary Tables 1–6, available online) associated with the effect allele of SNP *l*.

The primary PRSs were based on SNPs found to be associated with breast or ovarian cancer through genome-wide association studies (GWASs) in the general population. For breast cancer, we used the published PRSs for overall breast cancer, ER-positive breast cancer, and ER-negative breast cancer (8,20). In addition, we created updated PRSs based on findings from population-based association and fine-mapping studies reported before April 2015 (Supplementary Table 1, available online) (8,10,21–28). More details on the variant selection are provided in the Supplementary Methods (available online).

We developed an ovarian cancer PRS by including the most strongly associated variant from each region associated at a genome-wide statistical significance level with ovarian cancer risk in population-based studies or studies that combined population data and data from mutation carriers (Supplementary Table 2, available online) (9,23).

We also constructed secondary *BRCA1*- and *BRCA2*-specific PRSs that were based on all variants showing evidence of association in *BRCA1* and *BRCA2* carriers, using the results and weights from the *BRCA1-* and *BRCA2-*specific GWASs (11–13) (Supplementary Tables 3–6 and Supplementary Methods, available online). However, the studies that led to the identification of these variants were based on the same data set as the present analysis. Therefore, these *BRCA1-* and *BRCA2-*specific PRSs cannot be independently validated in the present analysis. To reduce the bias from overfitting, we also constructed and evaluated unweighted versions of these PRSs.

For the SNPs included in each PRS, we assessed whether there was evidence for pairwise interactions (Supplementary Methods, available online).

### Statistical Analysis

To account for the nonrandom sampling of mutation carriers with respect to disease status, the association of each PRS with breast or ovarian cancer risk was analyzed using a weighted cohort Cox regression with time to breast or ovarian cancer diagnosis, respectively, as the outcome (Supplementary Methods, available online) (29). We evaluated the associations of the breast cancer PRS (ie, overall breast cancer PRS, ER-positive PRS, and ER-negative PRS) with the risk for overall breast cancer for *BRCA1* and *BRCA2* mutation carriers. The ovarian cancer PRS was assessed for association with the risk of developing overall ovarian cancer for *BRCA1* and *BRCA2* mutation carriers. For these analyses, subjects were categorized into PRS percentile groups. To provide easily interpretable associations, the association analyses were repeated using continuous PRS predictors standardized to have mean 0 and variance 1. We assessed whether the hazard ratio per unit of the PRS varied with age by including a term for the interaction of the standardized PRS with age. We also fitted a Cox regression that included separate PRS effects by age group.

To evaluate the ability of the PRS to discriminate between individuals developing breast or ovarian cancer at different ages, we computed the rank Harrell’s c index (Supplementary Methods, available online) (30).

Absolute age-specific cumulative risks of developing breast or ovarian cancer at different percentiles of the standardized PRS were calculated according to the approach described previously (Supplementary Methods, available online) (15,31).

Analyses were carried out in R using GenABEL (32) and in STATA v13.1 (33). The associations of the continuous PRSs with breast or ovarian cancer risk were evaluated using one-sided statistical tests because we evaluated the directional hypothesis of increased cancer risk with a higher PRS. All other statistical tests were two-sided. Detailed methods are provided in the Supplementary Methods (available online).

## Results

### PRS Associations With Cancer Risks

Using data from 15 252 *BRCA1* and 8211 *BRCA2* carriers (Supplementary Table 7, available online), there was no evidence for interaction between any two variants involved in any of the PRSs after accounting for multiple testing (results not shown). All breast cancer PRSs derived from population-based study results (Supplementary Tables 1, available online) were statistically significantly associated with breast cancer risks for both *BRCA1* and *BRCA2* carriers (Table 1). Compared with the PRS developed by Mavaddat et al. (Supplementary Table 9, available online), the updated breast cancer PRS displayed slightly stronger associations in *BRCA1* carriers, but no improvements were seen in *BRCA2* carriers.

**Table 1.** Per-standard-deviation hazard ratios and 95% confidence intervals for the associations of polygenic risk scores with breast and ovarian cancer risk in *BRCA1* and *BRCA2* carriers*

PRS	No. of SNPs	*BRCA1* carriers		*BRCA2* carriers	
		HR (95% CI)	*P*†	HR (95% CI)	*P*†
Outcome: breast cancer					
Overall BC PRS	88	1.14 (1.11 to 1.17)	1.8x10^-18^	1.22 (1.17 to 1.28)	7.2x10^-20^
ER-positive BC PRS	87	1.11 (1.08 to 1.15)	3.5x10^-13^	1.22 (1.16 to 1.27)	4.0x10^-19^
ER-negative BC PRS	53	1.27 (1.23 to 1.31)	8.2x10^-53^	1.15 (1.10 to 1.20)	6.8x10^-10^
Outcome: ovarian cancer					
OC PRS	17	1.28 (1.22 to 1.34)	2.5x10^-26^	1.49 (1.34 to 1.65)	8.5x10^-14^

* The PRS created from the latest reported population-based study results were used. BC = breast cancer; CI = confidence interval; ER = estrogen receptor; HR = hazard ratio; OC = ovarian cancer; PRS = polygenic risk score; SNP = single-nucleotide polymorphism.

† *P* value for a one-sided test using a weighted cohort Cox regression with time to breast or ovarian cancer diagnosis, respectively, as the outcome.

The PRS for ER-negative breast cancer displayed the strongest association with breast cancer risk in *BRCA1* carriers (per standard deviation HR = 1.27, 95% confidence interval [CI] = 1.23 to 1.31, *P = *8.2×10^−53^) (Table 1). Smaller HR estimates in *BRCA1* breast cancer were seen for the PRS for overall breast cancer (HR = 1.14, 95% CI = 1.11 to 1.17, *P = *1.8×10^−18^) and ER-positive breast cancer (HR = 1.11, 95% CI =  1.08 to 1.15, *P = *3.5×10^−13^). In *BRCA2* carriers, the ER-negative breast cancer PRS displayed a smaller per SD HR for breast cancer risk (HR = 1.15, 95% CI =  1.10 to 1.20, *P = *6.8×10^−10^) compared with *BRCA1* carriers, whereas the overall breast cancer PRS (HR = 1.22, 95% CI =  1.17 to 1.28, *P = *7.2×10^−20^) and the ER-positive PRS (HR = 1.22, 95% CI = 1.16 to 1.27, *P = *4.0×10^−19^) displayed stronger associations. The subsequent breast cancer analyses focus on the updated ER-negative breast cancer PRS for *BRCA1* carriers and the updated overall breast cancer PRS for *BRCA2* carriers.

**Figure 1.**
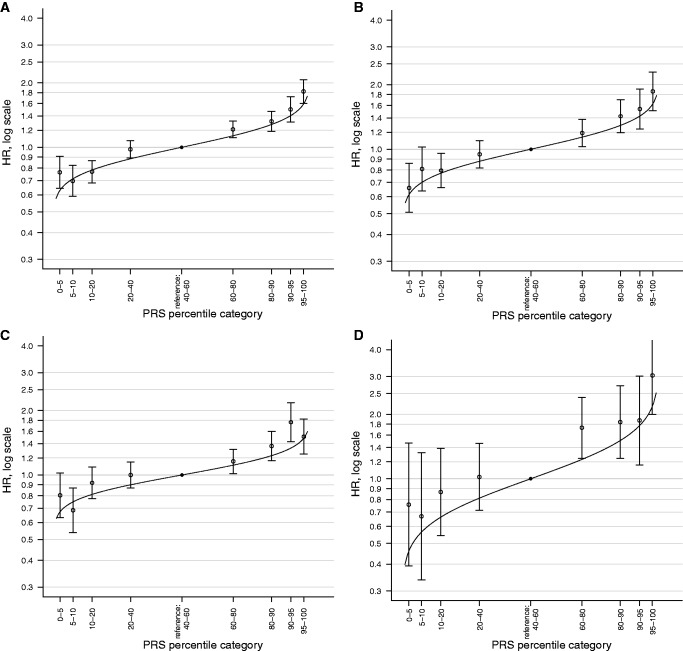
Hazard ratios (HRs) and 95% confidence intervals (**error bars**) for percentiles of the polygenic risk score (PRS) relative to the middle quintile. The estrogen receptor–negative breast cancer (BC) PRS **(A)** and the overall BC PRS **(C)** were used for breast cancer in *BRCA1* and *BRCA2* carriers, respectively, and the ovarian cancer (OC) PRS for the OC associations **(B, D)**. **Lines** denote the theoretical estimates under a multiplicative polygenic model with means and standard deviations of $\overset{-}{x}$ = 0.10 and SD = 0.41 for the ER-negative BC PRS, $\overset{-}{x}$ = 0.41 and SD = 0.50 for the overall BC PRS,$\;\overset{-}{x}$ = 0.47 and SD = 0.37 for the OC PRS.

Consistent with the above models, there were clear trends in risk by PRS for both *BRCA1* and *BRCA2* carriers when PRS was categorized by percentile (Table 2). The hazard ratio estimates were consistent with those predicted by the model, in which PRS was fitted as a continuous covariate (Figure 1).

**Table 2.** Proportion of samples and number of events in percentile categories of polygenic risk scores and their associations with breast and ovarian cancer risks*

Percentile category, %	*BRCA1* carriers		*BRCA2* carriers	
	No. of events (% samples in percentile category)	HR (95% CI)†	No. of events (% samples in percentile category)	HR (95% CI)†
Outcome: breast cancer				
0–5	222 (3.6)	0.76 (0.64 to 0.91)	138 (4.0)	0.80 (0.63 to 1.02)
5–10	250 (4.1)	0.70 (0.59 to 0.82)	142 (4.2)	0.68 (0.54 to 0.87)
10–20	551 (8.7)	0.77 (0.68 to 0.87)	340 (8.9)	0.92 (0.77 to 1.09)
20–40	1377 (18.7)	0.98 (0.89 to 1.07)	764 (18.8)	1.00 (0.87 to 1.15)
40–60	1534 (20.4)	1 (ref.)	793 (19.1)	1 (ref.)
60–80	1729 (21.0)	1.21 (1.11 to 1.33)	950 (21.2)	1.16 (1.02 to 1.32)
80–90	950 (11.0)	1.32 (1.19 to 1.47)	557 (11.4)	1.37 (1.17 to 1.60)
90–95	519 (5.8)	1.50 (1.31 to 1.72)	309 (5.8)	1.76 (1.43 to 2.17)
95–100	665 (6.7)	1.82 (1.61 to 2.07)	337 (6.7)	1.51 (1.25 to 1.82)
Outcome: ovarian cancer				
0–5	85 (4.7)	0.66 (0.51 to 0.86)	20 (4.8)	0.76 (0.39 to 1.47)
5–10	110 (5.3)	0.81 (0.64 to 1.02)	18 (5.3)	0.67 (0.34 to 1.32)
10–20	215 (10.5)	0.80 (0.66 to 0.96)	39 (10.4)	0.87 (0.54 to 1.39)
20–40	478 (20.9)	0.95 (0.82 to 1.10)	104 (20.4)	1.02 (0.71 to 1.46)
40–60	468 (19.9)	1 (ref.)	107 (20.4)	1 (ref.)
60–80	519 (19.5)	1.19 (1.03 to 1.38)	159 (19.5)	1.73 (1.25 to 2.40)
80–90	267 (9.3)	1.43 (1.20 to 1.70)	76 (9.1)	1.84 (1.24 to 2.72)
90–95	155 (4.9)	1.54 (1.24 to 1.91)	45 (4.8)	1.87 (1.16 to 3.02)
95–100	165 (5.1)	1.86 (1.51 to 2.29)	63 (5.4)	3.04 (2.00 to 4.61)

* The polygenic risk score (PRS) created from reported population-based study results were used. The percentile boundaries were derived assuming a normally distributed PRS. The estrogen receptor–negative breast cancer PRS was used for the associations with breast cancer risk in *BRCA1* carriers and overall breast cancer PRS in *BRCA2* carriers. CI = confidence interval; HR = hazard ratio.

† Hazard ratio from a weighted cohort Cox regression with time to breast or ovarian cancer diagnosis, respectively, as the outcome.

We also investigated whether the associations for the most strongly associated PRS differ by mutation type, as defined by the mutation functional effect (Supplementary Methods, available online). There was marginal evidence of an interaction between the breast cancer risk PRS and class 2 mutations in *BRCA2* mutation carriers (*P = *0.03, with a slightly higher HR estimate for the PRS for class 2 mutation carriers).

The population-based ovarian cancer PRS was strongly associated with ovarian cancer risk in *BRCA1* carriers with a per SD HR of 1.28 (95% CI = 1.22 to 1.34, *P = *2.5×10^−26^) (Table 1). The hazard ratio estimate was larger for ovarian cancer risk in *BRCA2* carriers (HR = 1.49, 95% CI = 1.34 to 1.65, *P = *8.5×10^−14^). When we compared the hazard ratio estimates against the hazard ratios predicted under a multiplicative polygenic model, only the hazard ratio estimate for *BRCA2* carriers for the 60% to 80% category was statistically significantly higher than the predicted value (Figure 1).

The unweighted *BRCA1*- and *BRCA2*-specific PRS for breast and ovarian cancer, constructed on the basis of association results in CIMBA, showed strong evidence of association with breast and ovarian cancer (Supplementary Table 10, available online).

### PRS x Age Interaction

There was evidence for a PRSxage interaction for the ER-negative breast cancer PRS for *BRCA1* carriers (*P = *3×10^−6^) and for the overall breast cancer PRS for *BRCA2* carriers (*P = *.01) (Table 3). In the ovarian cancer analysis, a statistically significant interaction with age was seen for the ovarian cancer PRS for *BRCA1* carriers (*P = *.003). Each of these PRSs showed stronger associations in younger age groups.

**Table 3.** Age-specific hazard ratio estimates for the PRS associations and HR estimates for a PRS x age interaction*

Age category, y	*BRCA1* carriers			*BRCA2* carriers		
	No. of events	HR per unit SD increase in the ER- PRS (95% CI)	*P*†	No. of events	HR per unit SD increase in the overall BC PRS (95% CI)	*P*†
Outcome: breast cancer						
18–39	4125	1.63 (1.52 to 1.74)	–	1731	1.65 (1.44 to 1.88)	–
40–49	2557	1.18 (1.13 to 1.23)	4.2 × 10^−15^	1587	1.22 (1.14 to 1.31)	8.5 × 10^−5^
50–59	846	1.14 (1.09 to 1.21)	.40	718	1.10 (1.02 to 1.19)	.05
≥60	269	1.20 (1.11 to 1.29)	.33	294	1.12 (1.03 to 1.23)	.75
Interaction HR		0.993 (0.990 to 0.996)	3.3 × 10^−6^		0.995 (0.991 to 0.999)	.01
Main effect PRS		1.69 (1.50 to 1.91)			1.55 (1.29 to 1.87)	
Outcome: ovarian cancer						
18–49	1258	1.55 (1.42 to 1.69)		172	3.05 (2.35 to 3.97)	
50–59	808	1.11 (1.05 to 1.18)	1.1 × 10^−9^	227	1.52 (1.26 to 1.84)	8.2 × 10^−6^
≥60	396	1.14 (1.06 to 1.21)	.67	232	1.21 (1.12 to 1.30)	.03
Interaction HR		0.992 (0.988 to 0.998)	.003		0.991 (0.979 to 1.00)	.11
Main effect PRS		1.83 (1.43 to 2.34)			2.48 (1.34 to 4.58)	

* The population-derived polygenic risk score (PRS) for estrogen receptor–negative breast cancer was used for the associations with breast cancer in *BRCA1* carriers and the overall breast cancer PRS in *BRCA2* carriers. *P* values relate to the difference in PRS association between each age group from the preceding younger group and to the interaction term. CI = confidence interval; HR = hazard ratio; PRS = polygenic risk score.

† *P* value for a two-sided test using a weighted cohort Cox regression with time to breast or ovarian cancer diagnosis, respectively, as the outcome.

### Discrimination

The ER-negative PRS had the highest value of Harrell's (c = 0.58, 95% CI =  0.57 to 0.59) for breast cancer in *BRCA1* carriers (Table 4). For breast cancer in *BRCA2* carriers, the highest values for Harrell's c were achieved by the population-based overall and ER-positive breast cancer PRSs (c = 0.56, 95% CI =  0.55 to 0.58, in each case). For ovarian cancer, the OC-PRS had a c of 0.58 (95% CI =  0.56 to 0.60) for *BRCA1* carriers and a c of 0.63 (95% CI =  0.60 to 0.67) for *BRCA2* carriers.

**Table 4.** Discrimination of population-derived polygenic risk scores for breast and ovarian cancer in *BRCA1* and *BRCA2* carriers*

PRS	Harrell’s c statistic (95% CI)	
	*BRCA1* carriers	*BRCA2* carriers
Discrimination for breast cancer		
Overall BC PRS	0.541 (0.530 to 0.551)	0.566 (0.551 to 0.581)
ER-positive BC PRS	0.532 (0.522 to 0.543)	0.566 (0.551 to 0.581)
ER-negative BC PRS	0.581 (0.571 to 0.592)	0.538 (0.523 to 0.553)
Discrimination for ovarian cancer		
OC PRS	0.579 (0.559 to 0.600)	0.628 (0.592 to 0.665)

* BC = breast cancer; CI = confidence interval; ER = estrogen receptor; OC = ovarian cancer; PRS = polygenic risk score.

### Predicted Absolute Risks by PRS Percentile

**Figure 2.**
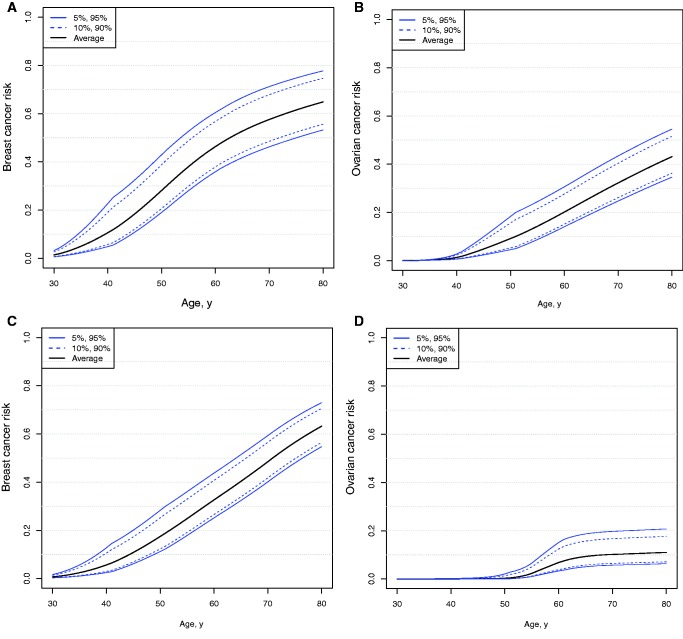
Predicted breast cancer risks by percentile of the polygenic risk scores (PRSs). The estrogen receptor–negative breast cancer PRS was used for *BRCA1* carriers **(A)** and the overall breast cancer PRS for *BRCA2* carriers **(C)**. Ovarian cancer risks are given by percentile of the ovarian cancer PRS in *BRCA1***(B)** and *BRCA2***(D)** carriers. Age-specific PRS associations were used to calculate these cumulative risks.

We used the age-specific hazard ratio estimates to compute absolute cumulative breast and ovarian cancer risks for mutation carrier by PRS percentiles (Figure 2). We used the updated ER-negative PRS to predict breast cancer risk for *BRCA1* carriers and the updated overall breast cancer PRS to predict breast cancer risk for *BRCA2* carriers. *BRCA1* carriers at the 10th percentile of the PRS had a risk of 21% of developing breast cancer by age 50 years and a 56% risk by age 80 years. In contrast, the *BRCA1* carriers at the 90th percentile of the PRS had a 39% breast cancer risk by age 50 years and 75% by age 80 years. The ovarian cancer risk was 6% by age 80 years for *BRCA2* carriers at the 10th percentile of the ovarian cancer PRS compared with 19% risk for those at the 90th percentile of PRS.

## Discussion

This is the first evaluation of the combined effects of all known common breast and ovarian cancer susceptibility loci on cancer risks for women who carry a *BRCA1* or *BRCA2* mutation. We found strong evidence of association with cancer risks for PRSs constructed using the results of population-based studies. These associations provide strong support for the hypothesis of a polygenic component for breast and ovarian cancer risks, respectively, that is largely shared between the general population and *BRCA1* and *BRCA2* mutation carriers. Moreover, the pattern of associations with the breast cancer subtype–specific PRS confirms the importance of tumor ER status (11). The PRS based on SNPs associated with ER-negative disease in the general population displayed a much stronger association with overall breast cancer risk for *BRCA1* carriers than the ER-positive PRS, consistent with the observation that the predominant tumor subtype in *BRCA1* carriers is ER negative (34,35). In contrast, the majority of tumors in *BRCA2* carriers tend to be ER positive. Consistent with this, the ER-positive PRS and the PRS for overall breast cancer constructed from general population data exhibited stronger associations than the ER-negative PRS in *BRCA2* carriers.

Using the overall, ER-positive, and ER-negative breast cancer PRSs developed by Mavaddat, the per SD hazard ratio estimates in mutation carriers were smaller than the corresponding per SD odds ratio estimates for breast cancer in the population-based study (20). These observations suggest that the relative extent by which the SNPs modify breast cancer risks in *BRCA1* and *BRCA2* mutation carriers is somewhat smaller than that in the general population, perhaps because a subset of SNPs do not combine multiplicatively with mutation status. Alternatively, these observations may reflect a difference in the design: Under a simple proportional hazards model, the predicted odds ratio is larger than the corresponding rate ratio (HR), but this difference is usually small (36). Moreover, some overestimation cannot be ruled out entirely for the per SD odds ratio estimates from the population-based study because of a winner’s curse effect. Interestingly, the hazard ratio estimate for the association of the ovarian cancer PRS with ovarian cancer risk was statistically significantly higher for *BRCA2* than for *BRCA1* mutation carriers. As a result, this PRS had also a higher discriminatory ability for ovarian cancer for *BRCA2* carriers compared with *BRCA1* mutation carriers.

Each of the most strongly associated PRSs displayed statistically significant interactions with age, with the exception of the ovarian cancer PRS in *BRCA2* carriers, such that the hazard ratio per unit PRS decreased with increasing age. One possible explanation for the observed interaction between age and the ER-negative breast cancer PRS in *BRCA1* mutation carriers could due to the use of the ER-negative breast cancer PRS from the general population to predict the risk of overall breast cancer risk for *BRCA1* mutation carriers. Although the majority of breast cancers in *BRCA1* mutation carriers are ER negative, the proportion of ER-negative breast tumors decreases with increasing age at diagnosis (35). If the population-based ER-negative PRSs were also associated primarily with ER-negative breast cancers in *BRCA1* mutation carriers, the ER-negative PRS would be more predictive of breast cancer in *BRCA1* carriers at younger ages. In contrast, in *BRCA2* carriers the proportion of ER-positive disease was found to decrease with increasing age at diagnosis (35). Therefore, the overall PRS from the general population, which is associated primarily with ER-positive breast cancers, may be more predictive of breast cancer in *BRCA2* mutation carriers at younger ages. Alternatively, it is possible that genetic risk modification has a stronger effect on developing early-onset breast cancer.

A limitation of the present study is our inability to take family history into account because this information was not available for the majority of samples. Although the tests of association remain valid, it was therefore not possible to investigate how the associations vary by family cancer history.

Overall, the discrimination achieved by the PRS investigated in the current study was moderate. The highest discrimination was achieved by the ovarian cancer PRS in *BRCA2* carriers. We found the overall breast cancer PRS to have somewhat lower discriminatory ability in mutation carriers compared with the general population (20). However, given the different study designs, ER tumor specificity in mutation carriers, and different measures of relative risk, these model performance estimates may not be directly comparable.

One possible explanation for the differences in the relative risk of the PRS between the mutation carriers and the population-based study is that not all variants identified in population-based studies are actually associated with risk in mutation carriers, perhaps as a result of functional redundancy (9). Conversely, variants that specifically modify risk in mutation carriers, examples of which have already been reported (13,18), would not be included in PRSs derived from population-based studies, and such variants might improve discrimination. On the other hand, because of the large sample sizes available in population-based studies, the SNP selection and the logOR estimates used as weights for these PRSs are likely to be more reliable than for PRSs based on mutation carriers. We also derived *BRCA1-* and *BRCA2*-specific PRSs that include variants discovered by population-based studies but only those showing evidence of association in mutation carriers. This approach makes use of the discovery power of population-based studies while accounting for possible mutation carrier–specific differences in associations. However, the SNP selection and weights were based on results from the same data set as that used in the present analysis. For this reason, we investigated the associations of mutation carrier–specific PRSs without weights to reduce the possible overfitting. An analysis in an independent sample of mutation carriers will be required to assess whether these mutation-specific PRSs outperform population-based PRSs.

The present study demonstrates that there are large differences in the absolute cancer risks between *BRCA1* and *BRCA2* mutation carriers with higher vs lower values of the PRS. These differences are much greater than those found in population-based studies (20,37) because the average risks conferred by *BRCA1* and *BRCA2* mutations are already high (17,18). The clinical management of healthy women with a *BRCA1* or *BRCA2* mutation involves a combination of frequent screening, risk-reducing surgery, and possibly chemoprevention (1), which can associated with substantial side effects. In particular, RRSO leads to premature menopause, is associated with increased morbidity, and has implications for family planning (38,39). Therefore, the timing of RRSO has to be carefully considered. There are no widely accepted risk thresholds for RRSO in mutation carriers: RRSO is recommended to all carriers on the basis of their average risk. The current National Comprehensive Cancer Network guidelines recommend RRSO for *BRCA1* carriers at age 35 to 40 years and *BRCA2* carriers at age 40 to 45 years (40). The average cumulative risk of ovarian cancer by age 40 years for *BRCA1* mutation carriers has been estimated as 2.8% (41). However, on the basis of our analyses, the cumulative risk of ovarian cancer for those at the lowest 1% of the PRS by age 40 years is predicted to be 0.7%, and 20% of *BRCA1* mutation carriers are predicted to have a risk of ovarian cancer of less than 1.3% by age 40 years. Therefore, the current results may be used to develop risk-based thresholds for RRSO recommendations. One possibility would be to assume that women with *BRCA1* mutations would not be offered RRSO until their cumulative risk of ovarian cancer approaches or exceeds 2.8%. A similar rule has recently been recommended for the counseling of women with mutations in moderate-risk genes (42). The ages at which women with *BRCA1* mutations would reach a cumulative risk of ovarian cancer of 2.8% are 48 years for those at the 1st percentile of the PRS, and 46, 45, 44, and 43 years for those at the 5th, 10th, 20th, and 30th percentiles of the PRS, respectively. For these women, deferring oophorectomy to these ages as opposed to the recommended age of 35 to 40 years may be preferable for childbearing and to avoid very early menopause. Another option would be to use risk-based thresholds defined for the general population. For example, a 10% lifetime risk of ovarian cancer is often cited as a recommended threshold for RRSO (43). Based on our results, *BRCA2* carriers at the 10th percentile of the ovarian cancer PRS have an estimated 6% lifetime risk and approximately 38% of *BRCA2* mutation carriers have a lifetime risk of ovarian cancer that is less than 10%. Women at this lower end of the risk spectrum might opt to delay RRSO to near or after natural menopause in order to avoid the harmful longer-term adverse effects of a surgically induced premature menopause, and this also provides a longer period for childbearing. Therefore, the PRS may be informative in guiding women with *BRCA1* and *BRCA2* mutations on the optimal timing of RRSO and can identify women at lower risk who may opt for less intensive interventions, such as salpingectomy with delayed oophorectomy.

Decisions in relation to breast cancer prevention could also be influenced by refined risk estimates. For example, the *BRCA1* carriers at the 90th percentile of the ER-negative breast cancer PRS had an estimated breast cancer risk of 19% by age 40 years and 39% by age 50 years, compared with 5% by age 40 years and 21% by age 50 years for carriers at the 10th percentile of the PRS. As with RRSO, there are currently no widely accepted risk thresholds for offering risk-reducing bilateral mastectomy (RRBM) for women with *BRCA1* and *BRCA2* mutations. However, studies in nonmutation carriers have shown that the uptake and timing of RRBM is directly related to the magnitude of breast cancer risk (44), and similar arguments may be applicable to mutation carriers. To provide comprehensive risk prediction, the PRS should be combined with other risk factors, including family history. Such a model would form the foundation for the development of risk-based clinical management guidelines for mutation carriers. In parallel, it will be necessary to perform risk communication studies to assess the acceptability of risk stratification in women with *BRCA1* and *BRCA2* mutations.

In conclusion, the results demonstrate that these PRSs could be useful in risk prediction for mutation carriers. Incorporating these PRSs into risk prediction models for *BRCA1* and *BRCA2* mutation carriers, together with other risk modifiers, may allow for more personalized risks for *BRCA1* and *BRCA2* mutation carriers and ultimately facilitate better management of mutation carriers.

## Funding

This work was supported by grants C12292/A11174 and C12292/A20861 from Cancer Research–UK. Funding for the iCOGS infrastructure came from the European Community's Seventh Framework Programme under grant agreement No. 223175 (HEALTH-F2-2009-223175; COGS), Cancer Research UK (C1287/A10118, C1287/A 10710, C12292/A11174, C1281/A12014, C5047/A8384, C5047/A15007, C5047/A10692, C8197/A16565), the National Institutes of Health (CA128978) and Post-Cancer GWAS initiative (1U19 CA148537, 1U19 CA148065 and 1U19 CA148112—the GAME-ON initiative), the Department of Defence (W81XWH-10-1-0341), the Canadian Institutes of Health Research (CIHR) for the CIHR Team in Familial Risks of Breast Cancer, Komen Foundation for the Cure, the Breast Cancer Research Foundation, and the Ovarian Cancer Research Fund. R01-CA122443 and P50-CA136393.

This work was supported by grant UM1 CA164920 from the National Cancer Institute. The content of this manuscript does not necessarily reflect the views or policies of the National Cancer Institute or any of the collaborating centers in the Breast Cancer Family Registry (BCFR), nor does mention of trade names, commercial products, or organizations imply endorsement by the US Government or the BCFR. Baltic Familial Breast Ovarian Cancer Consortium (BFBOCC) is partly supported by Lithuania (BFBOCC-LT): Research Council of Lithuania grant LIG-07/2012. Beth Israel Deaconess Medical Center (BIDMC) is supported by the Breast Cancer Research Foundation. BRCA gene mutations and breast cancer in South African women (BMBSA) was supported by grants from the Cancer Association of South Africa (CANSA) to Elizabeth J. van Rensburg. SLN was partially supported by the Morris and Horowitz Familes Endowed Professorship. This work was partially supported by Spanish Association against Cancer (AECC08), RTICC 06/0020/1060, FISPI08/1120, Mutua Madrileña Foundation (FMMA), and SAF2010-20493. City of Hope Clinical Cancer Genetics Community Network and the Hereditary Cancer Research Registry, supported in part by Award Number RC4CA153828 (PI: J. Weitzel) from the National Cancer Institute and the Office of the Director, National Institutes of Health. The content is solely the responsibility of the authors and does not necessarily represent the official views of the National Institutes of Health. Funds from Italian citizens who allocated the 5×1000 share of their tax payment in support of the Fondazione IRCCS Istituto Nazionale Tumori, according to Italian laws (INT-Institutional strategic projects ‘5×1000’) to SM and from FiorGen Foundation for Pharmacogenomics to LP. The CIMBA data management and data analysis were supported by Cancer Research–UK grants C12292/A11174, C12292/A20861 and C1287/A10118. SH is supported by a National Health and Medical Research Council (NHMRC) Program Grant to GCT. ACA is a Cancer Research–UK Senior Cancer Research Fellow. GCT is an NHMRC Senior Principal Research Fellow. This research has been cofinanced by the European Union (European Social Fund [ESF]) and Greek national funds through the Operational Program “Education and Lifelong Learning” of the National Strategic Reference Framework (NSRF) Research Funding Program of the General Secretariat for Research and Technology: SYN11_10_19 NBCA. Investing in knowledge society through the European Social Fund. The DKFZ (German Cancer Centre) study was supported by the DKFZ. EMBRACE is supported by Cancer Research UK grants C1287/A10118 and C1287/A11990. D. Gareth Evans and Fiona Lalloo are supported by a National Institute for Health Research (NIHR) grant to the Biomedical Research Centre, Manchester. The Investigators at The Institute of Cancer Research and The Royal Marsden National Health Service (NHS) Foundation Trust are supported by an NIHR grant to the Biomedical Research Centre at The Institute of Cancer Research and The Royal Marsden NHS Foundation Trust. Ros Eeles and Elizabeth Bancroft are supported by Cancer Research UK Grant C5047/A8385. Ros Eeles is also supported by NIHR support to the Biomedical Research Centre at The Institute of Cancer Research and The Royal Marsden NHS Foundation Trust. The authors acknowledge support from The University of Kansas Cancer Center (P30 CA168524) and the Kansas Bioscience Authority Eminent Scholar Program. AKG was funded by 5U01CA113916, R01CA140323, and by the Chancellors Distinguished Chair in Biomedical Sciences Professorship. The German Consortium of Hereditary Breast and Ovarian Cancer (GC-HBOC) is supported by the German Cancer Aid (grant no 109076, Rita K. Schmutzler) and by the Center for Molecular Medicine Cologne (CMMC). The study was supported by the Ligue Nationale Contre le Cancer, the Association “Le cancer du sein, parlons-en!” Award, the Canadian Institutes of Health Research for the “CIHR Team in Familial Risks of Breast Cancer” program, and the French National Institute of Cancer (INCa). CI received support from the Non-Therapeutic Subject Registry Shared Resource at Georgetown University (National Institutes of Health [NIH]/National Cancer Institute [NCI] grant P30-CA051008), the Fisher Center for Familial Cancer Research, and Swing For the Cure. Bruce Poppe is a senior clinical investigator of FWO. Was supported by a grant RD12/0036/0006 and 12/00539 from ISCIII (Spain), partially supported by European Regional Development FEDER funds. The Helsinki Breast Cancer Study (HEBCS) HEBCS was financially supported by the Helsinki University Hospital Research Fund, Academy of Finland (266528), the Finnish Cancer Society and the Sigrid Juselius Foundation. The HEBON study is supported by the Dutch Cancer Society grants NKI1998-1854, NKI2004-3088,and NKI2007-3756; the Netherlands Organization of Scientific Research grant NWO 91109024; the Pink Ribbon grant 110005; and the BBMRI grant NWO 184.021.007/CP46. HEBON thanks the registration teams of the Comprehensive Cancer Centre Netherlands and Comprehensive Centre South (together the Netherlands Cancer Registry) and PALGA (Dutch Pathology Registry) for part of the data collection. HRBCP is supported by The Hong Kong Hereditary Breast Cancer Family Registry and the Dr. Ellen Li Charitable Foundation, Hong Kong. The Hungarian Breast and Ovarian Cancer Study was supported by Hungarian Research grants KTIA-OTKA CK-80745, and OTKA K-112228 and the Norwegian EEA Financial Mechanism Hu0115/NA/2008-3/OP-9. ICO: contract grant sponsor: Asociación Española Contra el Cáncer, Spanish Health Research Fund; Carlos III Health Institute; Catalan Health Institute and Autonomous Government of Catalonia. Contract grant Nos.: ISCIIIRETIC RD06/0020/1051, RD12/0036/008, PI10/01422, PI10/00748, PI13/00285, PIE13/00022, 2009SGR290 and 2014SGR364. The IHCC was supported by Grant PBZ_KBN_122/P05/2004. The ILUH group was supported by the Icelandic Association “Walking for Breast Cancer Research” and by the Landspitali University Hospital Research Fund. This work was supported by the Canadian Institutes of Health Research (CIHR) for the “CIHR Team in Familial Risks of Breast Cancer” program, the Canadian Breast Cancer Research Alliance-grant No. 019511, and the Ministry of Economic Development, Innovation and Export Trade, grant No. PSR-SIIRI-701. IOVHBOCS is supported by Ministero della Salute and “5×1000” Istituto Oncologico Veneto grant. This study was in part supported by Liga Portuguesa Contra o Cancro. KConFab is supported by a grant from the National Breast Cancer Foundation, and previously by the National Health and Medical Research Council, the Queensland Cancer Fund, the Cancer Councils of New South Wales, Victoria, Tasmania and South Australia, and the Cancer Foundation of Western Australia. KOHBRA is supported by a grant from the National R&D Program for Cancer Control, Ministry for Health, Welfare and Family Affairs, Republic of Korea (1020350). MAYO is supported by NIH grants CA116167, CA128978, and CA176785; an National Cancer Institute (NCI) Specialized Program of Research Excellence (SPORE) in Breast Cancer (CA116201); a grant from the Breast Cancer Research Foundation; and a generous gift from the David F. and Margaret T. Grohne Family Foundation. Jewish General Hospital Weekend to End Breast Cancer, Quebec Ministry of Economic Development, Innovation and Export Trade. MODSQUAD was supported by MH CZ - DRO (MMCI, 00209805) and by the European Regional Development Fund and the State Budget of the Czech Republic (RECAMO, CZ.1.05/2.1.00/03.0101) to LF, and by Charles University in Prague project UNCE204024 (MZ). MSKCC is supported by grants from the Breast Cancer Research Foundation, the Robert and Kate Niehaus Clinical Cancer Genetics Initiative, and the Andrew Sabin Research Fund. 1R01 CA149429-01. The research of Drs. M. H. Greene and P. L. Mai was supported by the Intramural Research Program of the US National Cancer Institute, NIH, and by support services contracts NO2-CP-11019-50 and N02-CP-65504 with Westat, Inc., Rockville, MD. National Israeli Cancer Control Center (NICCC) is supported by Clalit Health Services in Israel. Some of its activities are supported by the Israel Cancer Association and the Breast Cancer Research Foundation (BCRF), NY. This work has been supported by the Russian Federation for Basic Research (grants 14-04-93959 and 15-04-01744). This study was supported by National Cancer Institute grants to the NRG Oncology Administrative Office and Tissue Bank (CA 27469), the NRG Oncology Statistical and Data Center (CA 37517), and NRG Oncology's Cancer Prevention and Control Committee (CA 101165). OSUCCG is supported by the Ohio State University Comprehensive Cancer Center. This work was supported by the ITT (Istituto Toscano Tumori) grants 2011-2013. Ministry of Science, Technology and Innovation, Ministry of Higher Education (UM.C/HlR/MOHE/06), and Cancer Research Initiatives Foundation. This project was partially funded through a grant by the Israel cancer association and the funding for the Israeli Inherited breast cancer consortium. SWE-BRCA collaborators are supported by the Swedish Cancer Society. UCHICAGO (University of Chicago) is supported by NCI Specialized Program of Research Excellence (SPORE) in Breast Cancer (CA125183), R01 CA142996, 1U01CA161032, and by the Ralph and Marion Falk Medical Research Trust, the Entertainment Industry Fund National Women's Cancer Research Alliance and the Breast Cancer research Foundation. OIO is an ACS Clinical Research Professor. Jonsson Comprehensive Cancer Center Foundation; Breast Cancer Research Foundation. UCSF Cancer Risk Program and Helen Diller Family Comprehensive Cancer Center. UKFOCR (UK and Gilda Radner Familial Ovarian Cancer Registries) was supported by a project grant from CRUK to Paul Pharoah. National Institutes of Health (NIH) (R01-CA102776 and R01-CA083855; Breast Cancer Research Foundation; Susan G. Komen Foundation for the cure, Basser Research Center for BRCA. Frieda G. and Saul F. Shapira BRCA-Associated Cancer Research Program; Hackers for Hope Pittsburgh. Kate Lawrenson is funded by Ovarian Cancer Research Fund (OCRF) grant No. 258807 and an Ann Schreiber Program of Excellence award from the Ovarian Cancer Research Fund (POE/USC/01.12). Janet Lee and Howard Shen are funded by National Institute of Health grant No. 5 U19 CA148112-02. Tassja Spindler is funded by National Institute of Health grant No. CA173531-01. Work was performed within the USC Norris Comprehensive Cancer Center, which is supported by a Cancer Center Support Grant (award No. P30 CA014089) from the National Cancer Institute. Victorian Cancer Agency, Cancer Australia, National Breast Cancer Foundation. Dr Karlan is funded by the American Cancer Society Early Detection Professorship (SIOP-06-258-01-COUN) and the National Center for Advancing Translational Sciences (NCATS), grant UL1TR000124.

## Notes

Authors: Karoline B. Kuchenbaecker, Lesley McGuffog, Daniel Barrowdale, Andrew Lee, Penny Soucy, Sue Healey, Joe Dennis, Michael Lush, Mark Robson, Amanda B. Spurdle, Susan J. Ramus, Nasim Mavaddat, Mary Beth Terry, Susan L. Neuhausen, Ute Hamann, Melissa Southey, Esther M. John, Wendy K. Chung, Mary B. Daly, Saundra S. Buys, David E. Goldgar, Cecilia M. Dorfling, Elizabeth J. van Rensburg, Yuan Chun Ding, Bent Ejlertsen, Anne-Marie Gerdes, Thomas V. O. Hansen, Susan Slager, Emily Hallberg, Javier Benitez, Ana Osorio, Nancy Cohen, William Lawler, Jeffrey N. Weitzel, Paolo Peterlongo, Valeria Pensotti, Riccardo Dolcetti, Monica Barile, Bernardo Bonanni, Jacopo Azzollini, Siranoush Manoukian, Bernard Peissel, Paolo Radice, Antonella Savarese, Laura Papi, Giuseppe Giannini, Florentia Fostira, Irene Konstantopoulou, Julian Adlard, Carole Brewer, Jackie Cook, Rosemarie Davidson, Diana Eccles, Ros Eeles, Steve Ellis, EMBRACE, Debra Frost, Shirley Hodgson, Louise Izatt, Fiona Lalloo, Kai-ren Ong, Andrew K. Godwin, Norbert Arnold, Bernd Dworniczak, Christoph Engel, Andrea Gehrig, Eric Hahnen, Jan Hauke, Karin Kast, Alfons Meindl, Dieter Niederacher, Rita Katharina Schmutzler, Raymonda Varon-Mateeva, Shan Wang-Gohrke, Barbara Wappenschmidt, Laure Barjhoux, Marie-Agnès Collonge-Rame, Camille Elan, GEMO Study Collaborators, Lisa Golmard, Emmanuelle Barouk-Simonet, Fabienne Lesueur, Sylvie Mazoyer, Joanna Sokolowska, Dominique Stoppa-Lyonnet, Claudine Isaacs, Kathleen B. M. Claes, Bruce Poppe, Miguel de la Hoya, Vanesa Garcia-Barberan, Kristiina Aittomäki, Heli Nevanlinna, Margreet G. E. M. Ausems, J. L. de Lange, Encarna B. Gómez Garcia, HEBON, Frans B. L. Hogervorst, Carolien M. Kets, Hanne E. J. Meijers-Heijboer, Jan C. Oosterwijk, Matti A. Rookus, Christi J. van Asperen, Ans M. W. van den Ouweland, Helena C. van Doorn, Theo A. M. van Os, Ava Kwong, Edith Olah, Orland Diez, Joan Brunet, Conxi Lazaro, Alex Teulé, Jacek Gronwald, Anna Jakubowska, Katarzyna Kaczmarek, Jan Lubinski, Grzegorz Sukiennicki, Rosa B. Barkardottir, Jocelyne Chiquette, Simona Agata, Marco Montagna, Manuel R. Teixeira, KConFab Investigators, Sue Kyung Park, Curtis Olswold, Marc Tischkowitz, Lenka Foretova, Pragna Gaddam, Joseph Vijai, Georg Pfeiler, Christine Rappaport-Fuerhauser, Christian F. Singer, Muy-Kheng M. Tea, Mark H. Greene, Jennifer T. Loud, Gad Rennert, Evgeny N. Imyanitov, Peter J. Hulick, John L. Hays, Marion Piedmonte, Gustavo C. Rodriguez, Julie Martyn, Gord Glendon, Anna Marie Mulligan, Irene L. Andrulis, Amanda Ewart Toland, Uffe Birk Jensen, Torben A. Kruse, Inge Sokilde Pedersen, Mads Thomassen, Maria A. Caligo, Soo-Hwang Teo, Raanan Berger, Eitan Friedman, Yael Laitman, Brita Arver, Ake Borg, Hans Ehrencrona, Johanna Rantala, Olufunmilayo I. Olopade, Patricia A. Ganz, Robert L. Nussbaum, Angela R. Bradbury, Susan M. Domchek, Katherine L. Nathanson, Banu K. Arun, Paul James, Beth Y. Karlan, Jenny Lester, Jacques Simard, Paul D. P. Pharoah, Kenneth Offit, Fergus J. Couch, Georgia Chenevix-Trench, Douglas F. Easton, Antonis C. Antoniou

Affiliations of authors: The Wellcome Trust Sanger Institute, Wellcome Trust Genome Campus, Hinxton, Cambridge, UK (KBK); Department of Public Health and Primary Care, University of Cambridge, Cambridge, UK (KBK, LM, DB, AL, JD, ML, NM, DFE, ACA); Genomics Center, Centre Hospitalier Universitaire de Québec Research Center and Laval University, Quebec City, Quebec, Canada (PS, JSi); Department of Genetics, QIMR Berghofer Medical Research Institute, Brisbane, Australia (SHe); Clinical Genetics, Service, Department of Medicine, Memorial Sloan Kettering Cancer Center, New York, NY (MR); Department of Genetics and Computational Biology, QIMR Berghofer Medical Research Institute, Brisbane, Australia (ABS, GCT); School of Women's and Children's Health, University of New South Wales, Australia; The Kinghorn Cancer Centre, Garvan Institute of Medical Research, 384 Victoria Street, Darlinghurst NSW 2010, Australia (SJR); Department of Epidemiology, Columbia University, New York, NY (MBT); Department of Population Sciences, Beckman Research Institute of City of Hope, Duarte, CA (SLN, YCD); Molecular Genetics of Breast Cancer, German Cancer Research Center, Heidelberg, Germany (UH); Genetic Epidemiology Laboratory, Department of Pathology, University of Melbourne, Parkville, VIC, Australia (MS); Department of Epidemiology, Cancer Prevention Institute of California, Fremont, CA (EMJ); Departments of Pediatrics and Medicine, Columbia University, New York, NY (WKC); Department of Clinical Genetics, Fox Chase Cancer Center, Philadelphia, PA (MBD); Department of Medicine, Huntsman Cancer Institute, Salt Lake City, UT (SSB); Department of Dermatology, University of Utah School of Medicine, Salt Lake City, UT (DEG); Cancer Genetics Laboratory, Department of Genetics, University of Pretoria, Arcadia, South Africa (CMD); Cancer Genetics Laboratory, Department of Genetics, University of Pretoria, Arcadia, South Africa (EJvR); Department of Oncology (BE), Department of Clincial Genetics (AMG), and Center for Genomic Medicine (TVOH), Rigshospitalet, Copenhagen University Hospital, Copenhagen, Denmark; Department of Health Sciences Research, Mayo Clinic, Rochester, MN (SS, EHal, CO); Human Genetics Group (JBe, AO) and Human Genotyping Unit, Human Cancer Genetics Program (JBe), Spanish National Cancer Centre , Madrid, Spain; Biomedical Network on Rare Diseases, CIBERER, Madrid, Spain (JBe, AO); City of Hope Clinical Cancer Genomics Community Research Network, Duarte, CA (NC, WL); Clinical Cancer Genetics, City of Hope, Duarte, California (JNW); IFOM, Institute of Molecular Oncology, Italian Foundation for Cancer Research, Milan, Italy (PP, VP); Cogentech Cancer Genetic Test Laboratory, Milan, Italy (VP); Centro di Riferimento Oncologico, IRCCS, Aviano, Italy (RDo); University of Queensland Diamantina Institute, Translational Research Institute, Brisbane, Australia (RDo); Division of Cancer Prevention and Genetics, Istituto Europeo di Oncologia, Milan, Italy (MB, BB); Unit of Medical Genetics (JAz, SMan, BPe) and Unit of Molecular Bases of Genetic Risk and Genetic Testing (PR), Department of Preventive and Predictive Medicine, Fondazione Istituto di Ricovero e Cura a Carattere Scientifico, Istituto Nazionale Tumori, Milan, Italy; Unit of Genetic Counselling, Medical Oncology Department, Istituto Nazionale Tumori Regina Elena, Rome, Italy (AS); Unit of Medical Genetics, Department of Biomedical, Experimental and Clinical Sciences, University of Florence, Florence, Italy (LP); Department of Molecular Medicine, University La Sapienza, Rome, Italy (GGi); Molecular Diagnostics Laboratory, Institute of Nuclear and Radiological Sciences and Technology, National Centre for Scientific Research Demokritos, Aghia Paraskevi Attikis, Athens, Greece (FF, IK); Yorkshire Regional Genetics Service, Chapel Allerton Hospital, Leeds, UK (JAd); Department of Clinical Genetics, Royal Devon and Exeter Hospital, Exeter, UK (CB); Sheffield Clinical Genetics Service, Sheffield Children’s Hospital, Sheffield, UK (JCo); Department of Clinical Genetics, South Glasgow University Hospitals, Glasgow, UK (RDa); University of Southampton Faculty of Medicine, Southampton University Hospitals NHS Trust, Southampton, UK (DE); Oncogenetics Team, The Institute of Cancer Research and Royal Marsden NHS Foundation Trust, London UK (RE); Centre for Cancer Genetic Epidemiology, Department of Public Health and Primary Care, University of Cambridge, Strangeways Research Laboratory, Cambridge, UK (SE, EMBRACE, DF); Medical Genetics Unit, St George's, University of London, London, UK (SHo); Clinical Genetics, Guy’s and St. Thomas’ NHS Foundation Trust, London, UK (LI); Genetic Medicine, Manchester Academic Health Sciences Centre, Central Manchester University Hospitals NHS Foundation Trust, Manchester, UK (FLa); West Midlands Regional Genetics Service, Birmingham Women’s Hospital Healthcare NHS Trust, Edgbaston, Birmingham, UK (KrO); Department of Pathology and Laboratory Medicine, University of Kansas Medical Center, Kansas City, KS (AKG); Department of Gynaecology and Obstetrics, University Hospital of Schleswig-Holstein, Campus Kiel, Christian-Albrechts University Kiel, Germany (NA); Institute of Human Genetics, University of Münster, Münster, Germany (BD); Institute for Medical Informatics, Statistics and Epidemiology, University of Leipzig, Germany (CEn); Centre of Familial Breast and Ovarian Cancer, Department of Medical Genetics, Institute of Human Genetics, University Würzburg, Germany (AG); Center for Hereditary Breast and Ovarian Cancer and Center for Integrated Oncology (CIO), Medical Faculty, University Hospital Cologne, Germany (EHah, JH, RKS, BW); Department of Gynaecology and Obstetrics, University Hospital Carl Gustav Carus, Technical University Dresden, Germany (KKas); Department of Gynaecology and Obstetrics, Division of Tumor Genetics, Klinikum Rechts der Isar, Technical University Munich, Germany (AM); Department of Gynaecology and Obstetrics, University Hospital Düsseldorf, Heinrich-Heine University Düsseldorf, Germany (DN); Institute of Human Genetics, Campus Virchov Klinikum, Charite Berlin, Germany (RVM); Department of Gynaecology and Obstetrics, University Hospital Ulm, Germany (SWG); Bâtiment Cheney D, Centre Léon Bérard, Lyon, France (LB); Lyon Neuroscience Research Center- CRNL, Inserm U1028, CNRS UMR5292, University of Lyon, Lyon, France (SMaz); Service de Génétique Biologique, CHU de Besançon, Besançon, France (ACR); Service de Génétique, Institut Curie, Paris, France (CEl, LG); Institut Curie, Department of Tumour Biology, Paris, France (GEMO, DSL); Institut Curie, INSERM U830, Paris, France (GEMO, DSL); Université Paris Descartes, Sorbonne Paris Cité, France (GEMO, DSL); Oncogénétique, Institut Bergonié, Bordeaux, France (EBS); Genetic Epidemiology of Cancer team, Inserm U900, Paris, France (FLe); Institut Curie, Paris, France (FLe); Mines ParisTech, Fontainebleau, France (FLe); Laboratoire de Génétique Médicale, Nancy Université, Centre Hospitalier Régional et Universitaire, Vandoeuvre-les-Nancy, France (JSo); Lombardi Comprehensive Cancer Center, Georgetown University, Washington, DC (CI); Center for Medical Genetics, Ghent University, Gent, Belgium (KBMC, BPo); Molecular Oncology Laboratory, Hospital Clinico San Carlos, El Instituto de Investigación Sanitaria del Hospital Clínico San Carlos, Madrid, Spain (MdlH, VGB); Department of Clinical Genetics, Helsinki University Hospital, HUS, Finland (KA); Department of Obstetrics and Gynecology, University of Helsinki and Helsinki University Hospital, Biomedicum Helsinki, HUS, Finland (HN); Department of Medical Genetics, University Medical Center Utrecht, Utrecht, the Netherlands (MGEMA); Department of Epidemiology (JLdL) and Coordinating Center, Hereditary Breast and Ovarian Cancer Research Group Netherlands (HEBON), Netherlands Cancer Institute, Amsterdam, the Netherlands (JLdL); Department of Clinical Genetics and GROW, School for Oncology and Developmental Biology, MUMC, Maastricht, the Netherlands (EBGG); Family Cancer Clinic, Netherlands Cancer Institute, Amsterdam, the Netherlands (FBLH); Department of Human Genetics, Radboud University Nijmegen Medical Centre, Nijmegen, the Netherlands (CMK); Department of Clinical Genetics, VU University Medical Centre, Amsterdam, the Netherlands (HEJMH); Department of Genetics, University Medical Center, Groningen University, Groningen, the Netherlands (JCO); Department of Epidemiology, Netherlands Cancer Institute, Amsterdam, the Netherlands (MAR); Department of Clinical Genetics Leiden University Medical Center Leiden, Leiden, the Netherlands (CJvA); Department of Clinical Genetics, Family Cancer Clinic, Erasmus University Medical Center, Rotterdam, the Netherlands (AMWvdO); Department of Gynaecology, Family Cancer Clinic, Erasmus MC Cancer Institute, Rotterdam, the Netherlands (HCvD); Department of Clinical Genetics, Academic Medical Center, Amsterdam, the Netherlands (TAMvO); Hong Kong Hereditary Breast Cancer Family Registry, Hong Kong (AK); Cancer Genetics Center, Hong Kong Sanatorium and Hospital, Hong Kong (AK); Department of Surgery, The University of Hong Kong, Hong Kong (AK); Department of Molecular Genetics, National Institute of Oncology, Budapest, Hungary (EO); Oncogenetics Group, Vall d’Hebron Institute of Oncology (VHIO), Universitat Autònoma de Barcelona, Vall d’Hebron University Hospital, Barcelona, Spain (OD); Genetic Counseling Unit, Hereditary Cancer Program, Institut d'Investigació Biomèdica de Girona, Catalan Institute of Oncology, Girona, Spain (JBr); Molecular Diagnostic Unit (CL) and Genetic Counseling Unit (AT), Hereditary Cancer Program, Bellvitge Biomedical Research Institute, Catalan Institute of Oncology, L'Hospitalet, Barcelona, Spain; Department of Genetics and Pathology, Pomeranian Medical University, Szczecin, Poland (JG, AJ, KKac, JLu, GS); Laboratory of Cell Biology, Department of Pathology, Reykjavik, Iceland (RBB); Biomedical Centre, Faculty of Medicine, University of Iceland, Reykjavik, Iceland (RBB); Unité de Recherche en Santé des Populations, Centre des Maladies du Sein Deschênes-Fabia, Hôpital du Saint-Sacrement, Québec, Canada (JCh); Immunology and Molecular Oncology Unit, Veneto Institute of Oncology IOV - IRCCS, Padua, Italy (SA, MM); Department of Genetics, Portuguese Oncology Institute of Porto, Porto, Portugal (MRT); Biomedical Sciences Institute (ICBAS), University of Porto, Porto, Portugal (MRT); Kathleen Cuningham Consortium for Research into Familial Breast Cancer, Peter MacCallum Cancer Center, Melbourne, Australia (KConFab); Department of Preventive Medicine, Department of Biomedical Science, and Cancer Research Institute, Seoul National University, Seoul, Korea (SKP); Program in Cancer Genetics, Departments of Human Genetics and Oncology, McGill University, Montreal, Quebec, Canada (MTi); Department of Cancer Epidemiology and Genetics, Masaryk Memorial Cancer Institute, Brno, Czech Republic (LF); Clinical Cancer Genetics Laboratory, Memorial Sloan Kettering Cancer Center, New York, NY (PG); Clinical Genetics Research Laboratory, Department of Medicine, Memorial Sloan Kettering Cancer Center, New York, NY (JV); Department of Gynecology and Gynecological Oncology, Comprehensive Cancer Center (GP), and Department of OB/GYN (CRF, CFS, MKMT), Medical University of Vienna, Vienna, Austria; Clinical Genetics Branch, Division of Cancer Epidemiology and Genetics, National Cancer Institute, National Institutes of Health, Rockville, MD (MHG, JTL); Clalit National Israeli Cancer Control Center and Department of Community Medicine and Epidemiology, Carmel Medical Center and B. Rappaport Faculty of Medicine, Haifa, Israel (GR); N. N. Petrov Institute of Oncology, St. Petersburg, Russia (ENI); Center for Medical Genetics, NorthShore University HealthSystem, University of Chicago Pritzker School of Medicine, Evanston, IL (PJH); The Ohio State University Comprehensive Cancer Center Arthur C. James Cancer Hospital and Richard J. Solove Research Institute Biomedical Research Tower, Columbus, OH (JLH); NRG Oncology, Statistics and Data Management Center, Roswell Park Cancer Institute, Buffalo, NY (MP); Division of Gynecologic Oncology, NorthShore University HealthSystem, University of Chicago, Evanston, IL (GCR); ANZGOG, NHMRC Clinical Trials Centre, Camperdown, Australia (JM); Ontario Cancer Genetics Network, Lunenfeld-Tanenbaum Research Institute, Mount Sinai Hospital, Toronto, Ontario, Canada (GGl); Laboratory Medicine Program, University Health Network, Toronto, Ontario, Canada (AMM); Department of Laboratory Medicine and Pathobiology, University of Toronto, Toronto, ON, Canada (AMM); Lunenfeld-Tanenbaum Research Institute, Mount Sinai Hospital, Toronto, Ontario, Canada (ILA); Departments of Molecular Genetics and Laboratory Medicine and Pathobiology, University of Toronto, Ontario, Canada (ILA); Division of Human Cancer Genetics, Departments of Internal Medicine and Molecular Virology, Immunology and Medical Genetics, Comprehensive Cancer Center, The Ohio State University, Columbus, OH (AET); Department of Clinical Genetics, Aarhus University Hospital, Aarhus N, Denmark (UBJ); Department of Clinical Genetics, Odense University Hospital, Odense C, Denmark (TAK, MTh); Section of Molecular Diagnostics, Clinical Biochemistry, Aalborg University Hospital, Aalborg, Denmark (ISP); Section of Genetic Oncology, Department of Laboratory Medicine, University and University Hospital of Pisa, Pisa Italy (MAC); Cancer Research Initiatives Foundation, Sime Darby Medical Centre, Subang Jaya, Malaysia (SHT); University Malaya Cancer Research Institute, University Malaya, Kuala Lumpur, Malaysia (SHT); The Institute of Oncology, Chaim Sheba Medical Center, Ramat Gan, Israel (RB); The Susanne Levy Gertner Oncogenetics Unit, Institute of Human Genetics, Chaim Sheba Medical Center, Ramat Gan, Israel (EF); Sackler Faculty of Medicine, Tel Aviv University, Ramat Aviv, Israel (EF); The Susanne Levy Gertner Oncogenetics Unit, Institute of Human Genetics, Chaim Sheba Medical Center, Ramat Gan, Israel (YL); Department of Oncology, Karolinska University Hospital, Stockholm, Sweden (BA); Department of Oncology, Clinical Sciences, Lund University and Skåne University Hospital, Lund, Sweden (AB); Department of Clinical Genetics, Lund University Hospital, Lund, Sweden (HE); Department of Clinical Genetics, Karolinska University Hospital L5:03, Stockholm, Sweden (JR); University of Chicago Medical Center, Chicago, IL (OIO); UCLA Schools of Medicine and Public Health, Division of Cancer Prevention and Control Research, Jonsson Comprehensive Cancer Center, Los Angeles, CA (PAG); Medical Sciences, University of California, San Francisco, San Francisco, CA (RLN); Department of Medicine, Abramson Cancer Center, Perelman School of Medicine at the University of Pennsylvania, Philadelphia, PA (ARB, SMD, KLN); Department of Breast Medical Oncology and Clinical Cancer Genetics Program, University Of Texas MD Andersson Cancer Center, Houston, TX (BKA); Familial Cancer Centre, Peter MacCallum Cancer Centre, Melbourne, VIC, Australia (PJ); Women's Cancer Program at the Samuel Oschin Comprehensive Cancer Institute, Cedars-Sinai Medical Center, Los Angeles, CA (BYK, JLe); Department of Oncology, University of Cambridge, Cambridge, UK (PDPP); Clinical Genetics Research Laboratory, Department of Medicine, Cancer Biology and Genetics, Memorial Sloan-Kettering Cancer Center, New York, NY 10044 (KO); Department of Laboratory Medicine and Pathology, and Health Sciences Research, Mayo Clinic, Rochester, MN (FJC). 

The funders had no role in the design of the study; the collection, analysis, or interpretation of the data; the writing of the manuscript; or the decision to submit the manuscript for publication.

Author contributions: KBK and ACA drafted the initial manuscript. KBK performed the statistical analyses. ACA, KBK, DFE, GCT, FC, and KO conceived and designed the study. LM and DB are the CIMBA database managers. GCT initiated and coordinates CIMBA. KBK, JD, and ML carried out the bioinformatics. All authors except KBK, DB, LM, ML, JD, and AL acquired phenotypic data and DNA samples or performed SNP genotyping. All authors read and approved the final manuscript. We wish to acknowledge Maggie Angelakos, Judi Maskiell, Gillian Dite, and Helen Tsimiklis. We wish to thank members and participants in the New York site of the Breast Cancer Family Registry for their contributions to the study. We wish to thank members and participants in the Ontario Familial Breast Cancer Registry for their contributions to the study. BFBOCC-LT acknowledges Vilius Rudaitis, Laimonas Griškevičius, and Ramūnas Janavičius (if not in the authorship). BFBOCC-LV acknowledges Drs. Janis Eglitis, Anna Krilova, and Aivars Stengrevics. BMBSA wishes to thank the families who contributed to the BMBSA study. We wish to thank Yuan Chun Ding and Linda Steele for their work in participant enrolment and biospecimen and data management. We thank Bent Ejlertsen for the recruitment and genetic counseling of participants. We thank Alicia Barroso, Rosario Alonso, and Guillermo Pita for their assistance; Alessandra Viel of the CRO Aviano National Cancer Institute, Aviano (PN), Italy; Laura Ottini of the “Sapienza” University, Rome, Italy; Liliana Varesco of the IRCCS AOU San Martino, IST Istituto Nazionale per la Ricerca sul Cancro, Genoa, Italy; Maria Grazia Tibiletti of the Ospedale di Circolo-Università dell'Insubria, Varese, Italy; Antonella Savarese of the Istituto Nazionale Tumori Regina Elena, Rome, Italy; Stefania Tommasi of the Istituto Nazionale Tumori “Giovanni Paolo II,” Bari, Italy; Irene Feroce of the Istituto Europeo di Oncologia, Milano, Italy; Aline Martayan of the Istituto Nazionale Tumori Regina Elena, Rome, Italy. The CIMBA data management and analysis is funded through Cancer Research–UK grant C12292/A11174. ACA is a Senior Cancer Research–UK Research Fellow. RE is supported by NIHR support to the Biomedical Research Centre at The Institute of Cancer Research and The Royal Marsden NHS Foundation Trust. We thank Ms. JoEllen Weaver and Dr. Betsy Bove for their technical support. Genetic Modifiers of Cancer Risk in BRCA1/2 Mutation Carriers (GEMO) study National Cancer Genetics Network “UNICANCER Genetic Group,” France. We wish to pay a tribute to Olga M. Sinilnikova, who with Dominique Stoppa-Lyonnet initiated and coordinated GEMO until she sadly passed away on the June 30, 2014, and to thank all the GEMO collaborating groups for their contribution to this study. GEMO Collaborating Centers are: Coordinating Centres, Unité Mixte de Génétique Constitutionnelle des Cancers Fréquents, Hospices Civils de Lyon, Centre Léon Bérard and Equipe “Génétique du cancer du sein,” Centre de Recherche en Cancérologie de Lyon: Olga Sinilnikova†, Sylvie Mazoyer, Francesca Damiola, Laure Barjhoux, Carole Verny-Pierre, Mélanie Léone, Nadia Boutry-Kryza, Alain Calender, Sophie Giraud; Service de Génétique Oncologique, Institut Curie, Paris: Dominique Stoppa-Lyonnet, Marion Gauthier-Villars, Bruno Buecher, Claude Houdayer, Etienne Rouleau, Lisa Golmard, Agnès Collet, Virginie Moncoutier, Muriel Belotti, Antoine de Pauw, Camille Elan, Catherine Nogues, Emmanuelle Fourme, Anne-Marie Birot; Institut Gustave Roussy, Villejuif: Brigitte Bressac-de-Paillerets, Olivier Caron, Marine Guillaud-Bataille; Centre Jean Perrin, Clermont–Ferrand: Yves-Jean Bignon, Nancy Uhrhammer; Centre Léon Bérard, Lyon: Christine Lasset, Valérie Bonadona, Sandrine Handallou. Centre François Baclesse, Caen: Agnès Hardouin, Pascaline Berthet, Dominique Vaur, Laurent Castera; Institut Paoli Calmettes, Marseille: Hagay Sobol, Violaine Bourdon, Tetsuro Noguchi, Audrey Remenieras, François Eisinger; CHU Arnaud-de-Villeneuve, Montpellier: Isabelle Coupier, Pascal Pujol. Centre Oscar Lambret, Lille: Jean-Philippe Peyrat, Joëlle Fournier, Françoise Révillion, Philippe Vennin†, Claude Adenis. Centre Paul Strauss, Strasbourg: Danièle Muller, Jean-Pierre Fricker; Institut Bergonié, Bordeaux: Emmanuelle Barouk-Simonet, Françoise Bonnet, Virginie Bubien, Nicolas Sevenet, Michel Longy; Institut Claudius Regaud, Toulouse: Christine Toulas, Rosine Guimbaud, Laurence Gladieff, Viviane Feillel; CHU Grenoble: Dominique Leroux, Hélène Dreyfus, Christine Rebischung, Magalie Peysselon; CHU Dijon: Fanny Coron, Laurence Faivre; CHU St-Etienne: Fabienne Prieur, Marine Lebrun, Caroline Kientz; Hôtel Dieu Centre Hospitalier, Chambéry: Sandra Fert Ferrer; Centre Antoine Lacassagne, Nice: Marc Frénay; CHU Limoges: Laurence Vénat-Bouvet; CHU Nantes: Capucine Delnatte; CHU Bretonneau, Tours: Isabelle Mortemousque; Groupe Hospitalier Pitié-Salpétrière, Paris: Florence Coulet, Chrystelle Colas, Florent Soubrier, Mathilde Warcoin; CHU Vandoeuvre-les-Nancy: Johanna Sokolowska, Myriam Bronner; CHU Besançon: Marie-Agnès Collonge-Rame, Alexandre Damette. Creighton University, Omaha, NE: Henry T. Lynch, Carrie L. Snyder. We wish to thank the technical support of Ilse Coene en Brecht Crombez. We acknowledge Alicia Tosar and Paula Diaque for their technical assistance. HEBCS would like to thank Dr. Kristiina Aittomäki, Taru A. Muranen, Drs. Carl Blomqvist and Kirsimari Aaltonen, and RNs Irja Erkkilä and Virpi Palola for their help with the HEBCS data and samples. The Hereditary Breast and Ovarian Cancer Research Group Netherlands (HEBON) consists of the following Collaborating Centers: Coordinating c\Center: Netherlands Cancer Institute, Amsterdam, NL: M. A. Rookus, F. B. L. Hogervorst, F. E. van Leeuwen, S. Verhoef, M. K. Schmidt, N. S. Russell, J. L. de Lange, R. Wijnands; Erasmus Medical Center, Rotterdam, NL: J. M. Collée, A. M. W. van den Ouweland, M. J. Hooning, C. Seynaeve, C. H. M. van Deurzen, I. M. Obdeijn; Leiden University Medical Center, NL: C. J. van Asperen, J. T. Wijnen, R. A. E. M. Tollenaar, P. Devilee, T. C. T. E. F. van Cronenburg; Radboud University Nijmegen Medical Center, NL: C. M. Kets, A. R. Mensenkamp; University Medical Center Utrecht, NL: M. G. E. M. Ausems, R. B. van der Luijt, C. C. van der Pol; Amsterdam Medical Center, NL: C. M. Aalfs, T. A. M. van Os; VU University Medical Center, Amsterdam, NL: J. J. P. Gille, Q. Waisfisz, H. E. J. Meijers-Heijboer; University Hospital Maastricht, NL: E. B. Gómez-Garcia, M. J. Blok; University Medical Center Groningen, NL: J. C. Oosterwijk, A. H. van der Hout, M. J. Mourits, G. H. de Bock; The Netherlands Foundation for the detection of hereditary tumours, Leiden, NL: H. F. Vasen; The Netherlands Comprehensive Cancer Organization (IKNL): S. Siesling, J. Verloop; The Dutch Pathology Registry (PALGA): L. I. H. Overbeek. The HEBON study is supported by the Dutch Cancer Society grants NKI1998-1854, NKI2004-3088, NKI2007-3756, the Netherlands Organization of Scientific Research grant NWO 91109024, the Pink Ribbon grants 110005 and 2014-187.WO76, the BBMRI grant NWO 184.021.007/CP46, and the Transcan grant JTC 2012 Cancer 12-054. HEBON thanks the registration teams of IKNL and PALGA for part of the data collection. We wish to thank Hong Kong Sanatorium and Hospital for their continued support. We wish to thank the Hungarian Breast and Ovarian Cancer Study Group members (Janos Papp, Tibor Vaszko, Aniko Bozsik, Timea Pocza, Judit Franko, Maria Balogh, Gabriella Domokos, Judit Ferenczi, Department of Molecular Genetics, National Institute of Oncology, Budapest, Hungary) and the clinicians and patients for their contributions to this study. We wish to thank the Oncogenetics Group (VHIO) and the High Risk and Cancer Prevention Unit of the University Hospital Vall d’Hebron. We wish to thank the ICO Hereditary Cancer Program team led by Dr. Gabriel Capella. We would like to thank Dr. Martine Dumont and Martine Tranchant for sample management and skillful technical assistance. JS is Chairholder of the Canada Research Chair in Oncogenetics. JS and PS were part of the QC and Genotyping coordinating group of iCOGS (BCAC and CIMBA). We wish to thank Drs. Ana Peixoto, Catarina Santos, Patrícia Rocha, and Pedro Pinto for their skillful contribution to the study. We wish to thank Heather Thorne, Eveline Niedermayr, all the KConFab research nurses and staff, the heads and staff of the Family Cancer Clinics, and the Clinical Follow Up Study (which has received funding from the NHMRC, the National Breast Cancer Foundation, Cancer Australia, and the US National Institute of Health) for their contributions to this resource, and the many families who contribute to kConFab. Modifier Study of Quantitative Effects on Disease (MODSQUAD): MODSQUAD acknowledges ModSQuaD members Csilla Szabo (National Human Genome Research Institute, National Institutes of Health, Bethesda, MD); Lenka Foretova and Eva Machackova (Department of Cancer Epidemiology and Genetics, Masaryk Memorial Cancer Institute and MF MU, Brno, Czech Republic); and Michal Zikan, Petr Pohlreich, and Zdenek Kleibl (Oncogynecologic Center and Department of Biochemistry and Experimental Oncology, First Faculty of Medicine, Charles University, Prague, Czech Republic). Anne Lincoln, Lauren Jacobs. We wish to thank the NICCC National Familial Cancer Consultation Service team led by Sara Dishon, the lab team led by Dr. Flavio Lejbkowicz, and the research field operations team led by Dr. Mila Pinchev. We thank the investigators of the Australia New Zealand NRG Oncology group. We wish to thank members and participants in the Ontario Cancer Genetics Network for their contributions to the study. Leigha Senter, Kevin Sweet, Julia Cooper, Caroline Craven, and Michelle O'Conor were instrumental in accrual of study participants, ascertainment of medical records, and database management. Samples were processed by the OSU Human Genetics Sample Bank. We would like to thank Yip Cheng Har, Nur Aishah Mohd Taib, Phuah Sze Yee, Norhashimah Hassan, and all the research nurses, research assistants, and doctors involved in the MyBrCa Study for assistance in patient recruitment, data collection, and sample preparation. In addition, we thank Philip Iau, Sng Jen-Hwei and Sharifah Nor Akmal for contributing samples from the Singapore Breast Cancer Study and the HUKM-HKL Study, respectively. The Malaysian Breast Cancer Genetic Study is funded by research grants from the Malaysian Ministry of Science, Technology and Innovation, Ministry of Higher Education (UM.C/HIR/MOHE/06), and charitable funding from Cancer Research Initiatives Foundation. SMC team wishes to acknowledge the assistance of the Meirav Comprehensive Breast Cancer Center team at the Sheba Medical Center for assistance in this study. Swedish scientists participating as SWE-BRCA collaborators are from Lund University and University Hospital: Åke Borg, Håkan Olsson, Helena Jernström, Karin Henriksson, Katja Harbst, Maria Soller, Ulf Kristoffersson; from Gothenburg Sahlgrenska University Hospital: Anna Öfverholm, Margareta Nordling, Per Karlsson, Zakaria Einbeigi; from Stockholm and Karolinska University Hospital: Anna von Wachenfeldt, Annelie Liljegren, Annika Lindblom, Brita Arver, Gisela Barbany Bustinza, Johanna Rantala; from Umeå University Hospital: Beatrice Melin, Christina Edwinsdotter Ardnor, Monica Emanuelsson; from Uppsala University: Hans Ehrencrona, Maritta Hellström Pigg, Richard Rosenquist; from Linköping University Hospital: Marie Stenmark-Askmalm, Sigrun Liedgren. We wish to thank Cecilia Zvocec, Qun Niu, physicians, genetic counselors, research nurses, and staff of the Cancer Risk Clinic for their contributions to this resource and the many families who contribute to our program. We thank Joyce Seldon MSGC, and Lorna Kwan, MPH, for assembling the data for this study. We would like to thank Dr. Robert Nussbaum and the following genetic counselors for participant recruitment: Beth Crawford, Kate Loranger, Julie Mak, Nicola Stewart, Robin Lee, Amie Blanco, and Peggy Conrad. And thanks to Ms. Salina Chan for her data management. We thank Simon Gayther, Carole Pye, Patricia Harrington, and Eva Wozniak for their contributions to the UKFOCR. We would like to thank Geoffrey Lindeman, Marion Harris, Martin Delatycki of the Victorian Familial Cancer Trials Group. We thank Sarah Sawyer and Rebecca Driessen for assembling this data and Ella Thompson for performing all DNA amplification.

## Supplementary Material

Supplementary Data
