# Supplementary material for: Evaluation of Polygenic Risk Scores for Breast and Ovarian Cancer Risk Prediction in BRCA1 and BRCA2 Mutation Carriers
Source: J Natl Cancer Inst. 2017 Mar 9;109(7):djw302. doi: 10.1093/jnci/djw302 (PMC5408990; doi:10.1093/jnci/djw302)
Supplement: Supplementary Data [file djw302_Supp.zip › 15-1761R2 Kuchenbaeker supp mat_111416.docx]

**SUPPLEMENTARY MATERIALS**

Evaluation of polygenic risk scores for breast and ovarian cancer risk prediction in *BRCA1* and *BRCA2* mutation carriers

Karoline B. Kuchenbaecker, PhD, et. al.

**Corresponding author:** Dr Antonis Antoniou, Strangeways Research Laboratory, Worts Causeway, Cambridge CB1 8RN, UK; email: antonis@srl.cam.ac.uk, tel.: +44 (0)1223 748630

# Supplementary Methods

## *Selection of SNPs and weights*

The primary PRS were based on SNPs found to be associated with breast or ovarian cancer through GWAS in the general population. For breast cancer, we used the PRS for overall breast cancer, ER-positive breast cancer and ER-negative breast cancer described by Mavaddat et al. (1) that was based on 77 SNPs associated with breast cancer risk in studies of the Breast Cancer Association Consortium (BCAC) (2).

In addition, we created updated PRS based on findings from population-based association and fine mapping studies reported by January 2015 (2-11). In general, variants were included in the updated breast cancer PRS if they displayed associations at genome-wide statistical significance (p<5x10^-8^) with breast cancer in population-based breast cancer case control studies that involved samples of European ancestry. The log odds ratios (ORs) from the respective logistic regressions reported in these studies were used as the weights for PRS construction (**Supplementary Tables 1-6**).

For regions where fine-mapping studies have identified more than one independent association signal at p<10^-4^ (6-8, 10, 11), a SNP representing each independent signal was included in the updated PRS and the weights were obtained from the multiple logistic regression analysis from the respective fine-mapping study that incorporated all variants as predictor variables in order to account for potential correlation between the variants.

Additional subtype-specific PRS were constructed on the basis of SNPs associated with oestrogen receptor- (ER) positive and negative disease, respectively. SNPs were included if they were associated at genome-wide statistical significance level with the risk of developing each breast cancer subtype. We also included SNPs that were associated with overall breast cancer at genome-wide statistical significance and the subtype-specific association was in the same direction, had p<0.05, and the association did not differ statistically significantly between ER subtypes (p for the difference in association >0.05). The weights used to compute these PRS were the log OR estimates from the logistic regression for the respective tumour subtype based on BCAC data (**Supplementary Table 1**).

So far no PRS has been published for ovarian cancer in the general population. Therefore, we created an ovarian cancer PRS by including the most strongly associated variant from each region associated at genome-wide statistical significance level with ovarian cancer risk in population-based studies or studies that combined population data and data from mutation carriers (6, 12). The published studies have not identified more than one independent association signal for any of the known ovarian cancer susceptibility regions. Therefore, for each locus we included the variant with the smallest p-value, based on the results of the Ovarian Cancer Association Consortium (OCAC). We used the PRS were the log ORs from the logistic regressions in OCAC for the weights in the PRS (**Supplementary Tables 1-6**).

In the main analysis we also included variants where the original discovery sample involved a contribution from the current data set of *BRCA1* and *BRCA2* mutation carriers. However, the weights were the log OR estimates from the population-based studies and therefore independent of the current data. In order to assess whether there was evidence of overestimation of the PRS associations, we carried out sensitivity analyses where we excluded SNPs that were associated at genome-wide statistical significance only when the population-based discovery sample was combined with data from *BRCA1* and/or *BRCA2* carriers (Supplementary Table 10). This affected the ER-negative breast cancer PRS (variants rs4577244 and rs8002929) and the ovarian cancer PRS (variants rs58722170, rs17329882, rs116133110, and rs635634). However, the potentially resulting bias from overfitting would be very minor because the weights for these variants were based on the independent population-based studies. The sensitivity analysis excluding those variants provided highly consistent results.

## *BRCA1- and BRCA2- specific PRS*

Four different scores for established genetic risk modifiers for mutation carriers were constructed (**Supplementary Tables 3-6**), one for breast cancer risk modifiers for *BRCA1* carriers, one for breast cancer risk modifiers for *BRCA2* carriers, one for ovarian cancer modifiers for *BRCA1* carriers and one for ovarian cancer modifiers for *BRCA2* carriers.

The information used to construct these scores was based on the association analyses described elsewhere (13-15). These PRS included all the variants that were associated at genome-wide statistical significance (p<5x10^-8^) in mutation carriers based on the CIMBA iCOGS data (separately for *BRCA1* and *BRCA2* mutation carriers). In addition, variants were also included in the PRS if they were associated at genome-wide statistical significance in population-based genome-wide association studies for breast or ovarian cancer, and were associated with risk in mutation carriers at p<0.05. The risk ratios used as weights in these PRS were the per-allele log HR estimate from the single SNP association analysis based on the retrospective likelihood estimation. The above PRS definition assumes that the risk ratios of all included variants combine multiplicatively on the risk scale. Where a genetic region has been shown to contain more than one independent association signal based on data from *BRCA1* or *BRCA2* mutation carriers (loci 5p15, 6q25, 10q26, 12p11, 12q24 and 16q12), the weights were log risk ratios estimates from a joint analysis that included two SNPs. For several loci, associations with both breast and ovarian cancer have been reported. The weights of these SNPs were assumed to be the log HRs from the competing risks analyses that simultaneously model the association with breast and with ovarian cancer.

## Tests for SNPxSNP interactions

For the SNPs included in each PRS we assessed whether there was evidence for pairwise interactions in their associations with breast or ovarian cancer risk in *BRCA1* or *BRCA2* carriers using Cox-regression within a survival analysis framework. For each pair of variants, the model included a term for the per-allele effect of each SNP and a term for their multiplicative interaction. The analyses were adjusted for birth cohort (decade) and stratified by sample country of origin. In order to account for non-independence between relatives in the sample, a robust variance approach was used (16). The analysis provides a valid test for the hypothesis of interaction effect (17). Due to multiple testing, we considered a Bonferroni adjusted statistical significance threshold. The adjusted p-value thresholds ranged from p<4x10^-3^ to p<1x10^-5^, depending on the number of SNPs for different PRS.

## Outcomes

As outcomes, we considered breast and ovarian cancer in *BRCA1* and *BRCA2* carriers. In the breast cancer analysis, each mutation carrier was followed until breast or ovarian cancer diagnosis, bilateral prophylactic mastectomy, or age at last observation whichever occurred first. Of the 15,252 *BRCA1* carriers, 7,797 were considered affected with breast cancer and of the 8,211 *BRCA2* carriers 4,330 considered affected in this analysis. In the ovarian cancer analysis, mutation carriers were followed until the age of ovarian cancer diagnosis, age at risk-reducing salpingo-oophorectomy (RRSO) or age at last observation. Breast cancer diagnosis was not considered as a censoring event in the ovarian cancer analysis. A total of 2,462 *BRCA1* and 631 *BRCA2* mutation carriers diagnosed with ovarian cancer were considered affected in this analysis.

## PRS associations with breast and ovarian cancer risk

To account for the non-random sampling of mutation carriers with respect to their disease status, the association of each PRS with breast or ovarian cancer risk was analysed using a weighted cohort Cox regression with time to breast or ovarian cancer diagnosis, respectively, as the outcome (18, 19). Population-based incidences (20) were used to compute the weights separately for breast and ovarian cancer. A robust variance approach was used to account for related individuals in the sample. The analyses were stratified by country of residence. The USA and Canada strata were further subdivided by reported Ashkenazi Jewish ancestry. The analyses were adjusted for year of birth using a set of indicator variables (<1945, 1945-1955, 1955-1965, 1965-1975, >1975) to account for increased incidence rates in more recent birth cohorts (21).

For these analyses, the PRS were categorised into percentile groups: 0-5%, 5-10%, 10-20%, 20-40%, 40-60%, 60-80%, 80-90%, 90-95%, 95-100%. Since the sampling scheme in CIMBA is dependent on outcome (age and phenotype), which could affect the observed PRS distribution, we derived the boundaries for the percentile groups using the theoretical PRS distribution by assuming the PRS are normally distributed with a variance as estimated using our sample. The mean was calculated as

$$\bar{PRS}=\sum_{l=1}^{k} 2p_{i}\left( 1-p_{i} \right)\beta_{i}+p_{i}^{2}2\beta_{i}$$

where p_i_ is the minor allele frequency of SNP i retrieved from the European subset of the 1000 Genomes Project data. For each percentile category a hazard ratio (HR) was estimated relative to the middle category (40-60%) as the reference category.

In order to provide more easily interpretable association results and make the results from different analyses comparable, the association analyses were repeated using PRS predictors that was standardised to have mean 0 and variance 1:

$$z_{i}=\left( {PRS}_{i}-\bar{PRS} \right)/{SD}$$

where PRS_i_ is the PRS value of individual i, $\bar{PRS}$ is the mean PRS value and SD the standard deviation. The resulting estimate for the regression coefficient from the weighted Cox regression analysis using the standardised PRS could then be interpreted as the log HR per standard deviation of the PRS. This model assumes that the log-hazard varies linearly with the PRS. In order to assess this assumption, we compared the HR estimates for the discrete percentile categories with the predicted estimates based on the HR from the continuous model for the respective quantile. The PRS association analyses used one-tailed tests because we hypothesised that carrying higher numbers of risk alleles was associated with higher cancer risks.

## *Analysis by mutation type*

We carried out a sensitivity analysis where we assessed the associations of the PRS with breast cancer risk by type of the BRCA1 or BRCA2 mutation defined by their functional effect. This was done by including an interaction term for the PRS by mutation class in the association analysis described in the main methods. Class 1 mutations comprised loss-of-function mutations, expected to result in a reduced transcript or protein level due to mRNA nonsense-mediated decay (NMD) and/or degradation or instability of truncated proteins, translation re-initiation but no production of stable protein, or the absence of expression because of the deletion of transcription regulatory regions. Class 2 mutations were those likely to generate potentially stable mutant proteins that might have dominant negative action, partially preserved normal function, or loss of function. Class 2 mutations include missense substitutions, in-frame deletions and insertion, as well as truncating mutations with premature stop codons occurring in the last exon. Mutations, whose consequences at transcript or protein level could not be inferred, were not considered for this classification. To assess whether there are differences in the associations by mutation type we fitted an interaction term between the PRS and mutation class.

## *Age-specific PRS*

We assessed whether the HR per unit of the PRS varied by age by including a term for the interaction of the standardised PRS with age. We also fitted a Cox regression that included separate PRS effects by age group (<40, 40-49, 50-59 and ≥60 years in the breast cancer analysis and <50, 50-59 and ≥60 years in the ovarian cancer analysis).

## Assessing discrimination

In order to evaluate the ability of the PRS to discriminate between individuals developing breast or ovarian cancer at different ages, rank parameters quantifying the ordinal predictive power in a survival analysis setting were computed (22-24). Based on the log HR coefficients from the country-stratified weighted cohort analyses for the continuous PRS, Somers' D was computed using the somersd package in STATA (25). Somers' D represents the difference between the probability of concordance, that for a given pair the person with the higher PRS value was diagnosed with cancer at younger age, and the probability of discordance (26). For any pair of samples, the person who stays breast cancer free for longer is Dx100 % more likely to have the lower of the two PRS values. The estimate for Somers' D is related to Harrell's c index through c=(D+1)/2. The area under the receiver operator characteristic curve (AUC) for binary outcome variables is a special case of Harrell's c. It can take values between 0 and 1 with values of 0.5 indicating that the PRS has no predictive value and values closer to 1 indicating better discrimination.

## Absolute risks of breast and ovarian cancer by PRS

Age specific cumulative risks of developing breast or ovarian cancer at different percentiles of the standardised PRS were calculated according to the approach described by Antoniou et al. and Al Olama et al. (27, 28). The average age-specific incidences over all possible PRS categories were constrained to agree with published estimates of the average breast and ovarian cancer incidences for *BRCA1* and *BRCA2* mutation carriers (21). The PRS were categorised into the 0-5%, 5-10%, 10-20%, 20-40%, 40-60%, 60-80%, 80-90%, 90-95% and 95-100% percentile groups as described above.

We repeated this analysis using age-specific HRs per SD of the PRS from the model described above (for <40, 40-49, 50-59, ≥60 years for breast and <50, 50-59, ≥60 for ovarian cancer). Since the risk distribution is continuous in this case, we implemented the constraints to the incidence by approximating the normally distributed PRS by a binomial distribution with n=2000. Absolute risks were calculated for specific percentiles and reported as the lower or upper limit for individuals at that percentile or a more extreme PRS value, assuming that individuals with higher PRS values have higher risks.

**Supplementary Tables**

*Supplementary Tables 1-6 are available for download as a separate .xls file.*

**Supplementary Table 1.** Variants included in the polygenic risk scores for overall, oestrogen receptor positive (ER+) and oestrogen receptor negative (ER-) breast cancer (BC) based on the latest general-population study results.

**Supplementary Table 2.** Variants included in the polygenic risk scores for ovarian cancer based on the latest general-population study results.

**Supplementary Table 3.** Variants included in the *BRCA1*-carriers-specific polygenic risk score for breast cancer.

**Supplementary Table 4.** Variants included in the *BRCA2*-carriers-specific polygenic risk score for breast cancer.

**Supplementary Table 5.** Variants included in the *BRCA1*-carriers-specific polygenic risk score for ovarian cancer.

**Supplementary Table 6.** Variants included in the *BRCA2*-carriers-specific polygenic risk score for ovarian cancer.

Supplementary Table 7: Year of birth, censoring age and mutation class of breast cancer (BC) and ovarian cancer (OC) affected and unaffected BRCA1 and BRCA2 mutation carriers

| **Variable** | **BRCA1 mutation carriers** | | | | **BRCA2 mutation carriers** | | | |
| --- | --- | --- | --- | --- | --- | --- | --- | --- |
|  | **Breast Cancer Analysis** | | **Ovarian Cancer Analysis** | | **Breast Cancer Analysis** | | **Ovarian Cancer Analysis** | |
|  | **unaffected** | **affected** | **unaffected** | **affected** | **unaffected** | **affected** | **unaffected** | **affected** |
| N | 7455 | 7797 | 12790 | 2462 | 3881 | 4330 | 7580 | 631 |
| Year of Birth |  |  |  |  |  |  |  |  |
| ≤1920 | 39 | 49 | 75 | 13 | 29 | 43 | 57 | 15 |
| 1921-1930 | 146 | 209 | 223 | 132 | 104 | 183 | 223 | 64 |
| 1931-1940 | 411 | 602 | 641 | 372 | 272 | 485 | 574 | 183 |
| 1941-1950 | 978 | 1653 | 1812 | 819 | 571 | 1030 | 1370 | 231 |
| 1951-1960 | 1671 | 2365 | 3278 | 758 | 810 | 1380 | 2078 | 112 |
| >1960 | 4210 | 2919 | 6761 | 368 | 2095 | 1209 | 3278 | 26 |
| Median censoring age (interquartile range) | 41  (33-50) | 40  (35-47) | 42  (35-51) | 50  (45-57) | 43  (34-53) | 43  (37-50) | 46  (38-55) | 57  (50-64) |
| ≤40 | 3452 | 3986 | 5443 | 274 | 1640 | 1653 | 2488 | 24 |
| 41-50 | 2118 | 2627 | 4051 | 942 | 1032 | 1620 | 2408 | 142 |
| 51-60 | 1231 | 910 | 2116 | 826 | 686 | 747 | 1558 | 222 |
| >60 | 654 | 274 | 118 | 420 | 523 | 310 | 1126 | 243 |
| Mutation class* |  |  |  |  |  |  |  |  |
| Class I | 5295 | 5044 | 8531 | 1808 | 3623 | 3999 | 7029 | 593 |
| Class II | 1652 | 2037 | 3219 | 470 | 108 | 130 | 226 | 12 |
| Class III | 508 | 716 | 1040 | 184 | 150 | 201 | 325 | 26 |

* Mutation class I: unstable/no protein, class II: stable mutant protein, class III: consequence unknown. (See the **Supplementary Methods** for a full description).

Supplementary Table 8. Means of population-derived polygenic risk scores (PRS) in breast (BC) or ovarian cancer unaffected and affected samples in the entire sample*

| **PRS** | **No. of SNPs** | ***BRCA1* carriers** | | | ***BRCA2* carriers** | | |
| --- | --- | --- | --- | --- | --- | --- | --- |
|  |  | **mean unaffected** | **mean affected** | **SD** | **mean unaffected** | **mean affected** | **SD** |
| Breast cancer |  |  |  |  |  |  |  |
| Overall BC PRS by Mavaddat et al.(1) | 77 | 0.564 | 0.628 | 0.448 | 0.571 | 0.679 | 0.449 |
| Estrogen-receptor-positive BC PRS by Mavaddat et al.(1) | 77 | 0.547 | 0.601 | 0.499 | 0.548 | 0.668 | 0.501 |
| Estrogen-receptor-negative BC PRS by Mavaddat et al.(1) | 77 | 0.482 | 0.574 | 0.392 | 0.517 | 0.569 | 0.393 |
| Our updated BC PRS | 88 | 0.392 | 0.466 | 0.500 | 0.412 | 0.532 | 0.506 |
| Our updated oestrogen-receptor-positive BC PRS | 87 | 0.411 | 0.481 | 0.556 | 0.426 | 0.557 | 0.565 |
| Our updated oestrogen-receptor-positive PRS | 53 | 0.0985 | 0.202 | 0.412 | 0.133 | 0.191 | 0.421 |
| Ovarian cancer |  |  |  |  |  |  |  |
| Ovarian cancer PRS | 17 | 0.452 | 0.517 | 0.373 | 0.454 | 0.595 | 0.377 |

Supplementary Table 9. Association of the published population-based polygenic risk scores (PRS) by Mavaddat et al.(1) based on 77 breast cancer (BC) susceptibility variants with breast cancer risk in *BRCA1* and *BRCA2* carriers*

| **PRS category/Discovery set** | ***BRCA1* carriers** | | ***BRCA2* carriers** | |
| --- | --- | --- | --- | --- |
|  | **HR (95%CI)** | **P†** | **HR (95%CI)** | **P†** |
| Overall BC | 1.13 (1.10-1.16) | 1.5x10^-16^ | 1.22 (1.17-1.28) | 3.8x10^-20^ |
| ER+ BC | 1.09 (1.06-1.12) | 2.5x10^-9^ | 1.22 (1.17-1.27) | 5.0x10^-20^ |
| ER- BC | 1.24 (1.21-1.28) | 8.1x10^-48^ | 1.13 (1.08-1.18) | 2.8x10^-8^ |

* The hazard ratios (HR) are per standard deviation of the score. CI=confidence interval; BC=breast cancer; ER=estrogen receptor.

† P-value for a two-sided test using a weighted cohort Cox-regression with time to breast or ovarian cancer diagnosis, respectively, as the outcome

Supplementary Table 10. Association of unweighted *BRCA1*- and *BRCA2*-carrier-specific breast (BC) and ovarian cancer (OC) polygenic risk scores (PRS) with breast and ovarian cancer risk in *BRCA1* and *BRCA2* carriers

| **PRS category/Discovery set** | **No. of SNPs** | ***BRCA1* carriers** | | ***BRCA2* carriers** | |
| --- | --- | --- | --- | --- | --- |
|  |  | **HR (95%CI)*** | **P†** | **HR (95%CI)*** | **P†** |
| Breast cancer |  |  |  |  |  |
| *BRCA1* carriers BC‡ | 24 | 1.29 (1.26-1.34) | 8.1x10^-65^ | 1.14 (1.09-1.19) | 2.6x10^-9^ |
| *BRCA2* carriers BC§ | 20 | 1.12 (1.09-1.16) | 9.3x10^-16^ | 1.29 (1.23-1.35) | 1.1x10^-30^ |
| Ovarian cancer |  |  |  |  |  |
| *BRCA1* carriers OC‡ | 13 | 1.30 (1.25-1.36) | 1.0x10^-31^ | 1.36 (1.23-1.51) | 2.3x10^-9^ |
| *BRCA2* carriers OC§ | 8 | 1.21 (1.15-1.26) | 2.5x10^-16^ | 1.49 (1.34-1.65) | 7.0x10^-14^ |

* The hazard ratios (HR) are per standard deviation of the score. BC=breast cancer; CI=confidence interval; OC=ovarian cancer.

† P-value for a two-sided test using a weighted cohort Cox-regression with time to breast or ovarian cancer diagnosis, respectively, as the outcome

‡ Association of the *BRCA1* CIMBA-based PRS. The *BRCA1* carrier data formed the discovery set so the association reported here for *BRCA1* carriers does not represent an independent validation

§ Association of the *BRCA2* CIMBA-based PRS. The *BRCA2* carrier data formed the discovery set so the association reported here for *BRCA2* carriers does not represent an independent validation

Supplementary Table 11. Per-standard-deviation hazard ratios (HR) and 95% confidence intervals (CI) for the associations of polygenic risk scores (PRS) with breast (BC) and ovarian cancer (OC) risk in *BRCA1* and *BRCA2* carriers*****

| **PRS** | **No. of SNPs** | ***BRCA1* carriers** | | ***BRCA2* carriers** | |
| --- | --- | --- | --- | --- | --- |
|  |  | **HR (95%CI)** | **P†** | **HR (95%CI)** | **P†** |
| Outcome: Breast cancer | | | | | |
| ER-negative BC PRS | 51 | 1.25 (1.22-1.29) | 1.0x10^-48^ | 1.15 (1.10-1.20) | 3.0x10^-10^ |
| Outcome: Ovarian cancer | | | | | |
| OC PRS | 17 | 1.26 (1.20-1.31) | 1.7x10^-22^ | 1.44 (1.30-1.60) | 2.3x10^-12^ |

* The PRS were created from the latest reported population-based study results but excluded variants discovered through combined analysis involving the present validation data (rs4577244 and rs8002929 for oestrogen receptor (ER)-negative BC PRS; rs58722170, rs17329882, rs116133110, and rs635634 for the OC PRS).

† P-value for a two-sided test using a weighted cohort Cox-regression with time to breast or ovarian cancer diagnosis, respectively, as the outcome.

**References**

1. Mavaddat N, Pharoah PD, Michailidou K*, et al.* Prediction of breast cancer risk based on profiling with common genetic variants. J Natl Cancer Inst 2015;107(5).

2. Michailidou K, Hall P, Gonzalez-Neira A*, et al.* Large-scale genotyping identifies 41 new loci associated with breast cancer risk. Nat Genet 2013;45(4):353-61, 361e1-2.

3. Michailidou K, Beesley J, Lindstrom S*, et al.* Genome-wide association analysis of more than 120,000 individuals identifies 15 new susceptibility loci for breast cancer. Nat Genet 2015;47(4):373-80.

4. Lin WY, Camp NJ, Ghoussaini M*, et al.* Identification and characterization of novel associations in the CASP8/ALS2CR12 region on chromosome 2 with breast cancer risk. Hum Mol Genet 2015;24(1):285-98.

5. Ghoussaini M, Edwards SL, Michailidou K*, et al.* Evidence that breast cancer risk at the 2q35 locus is mediated through IGFBP5 regulation. Nat Commun 2014;4:4999.

6. Bojesen SE, Pooley KA, Johnatty SE*, et al.* Multiple independent variants at the TERT locus are associated with telomere length and risks of breast and ovarian cancer. Nat Genet 2013;45(4):371-84, 384e1-2.

7. French JD, Ghoussaini M, Edwards SL*, et al.* Functional variants at the 11q13 risk locus for breast cancer regulate cyclin D1 expression through long-range enhancers. Am J Hum Genet 2013;92(4):489-503.

8. Meyer KB, O'Reilly M, Michailidou K*, et al.* Fine-scale mapping of the FGFR2 breast cancer risk locus: putative functional variants differentially bind FOXA1 and E2F1. Am J Hum Genet 2013;93(6):1046-60.

9. Southey MC, Goldgar DE, Winqvist R*, et al.* PALB2, CHEK2 and ATM rare variants and cancer risk: data from COGS. J Med Genet 2016; 10.1136/jmedgenet-2016-103839.

10. Dunning AM, Michailidou K, Kuchenbaecker KB*, et al.* Breast cancer risk variants at 6q25 display different phenotype associations and regulate ESR1, RMND1 and CCDC170. Nat Genet 2016;48(4):374-86.

11. BCAC. *iCOGS summary results*. <http://bcac.ccge.medschl.cam.ac.uk/bcacdata/icogs-summary-results/>.

12. Kuchenbaecker KB, Ramus SJ, Tyrer J*, et al.* Identification of six new susceptibility loci for invasive epithelial ovarian cancer. Nat Genet 2015;47(2):164-71.

13. Couch FJ, Wang X, McGuffog L*, et al.* Genome-wide association study in BRCA1 mutation carriers identifies novel loci associated with breast and ovarian cancer risk. PLoS Genet 2013;9(3):e1003212.

14. Gaudet MM, Kuchenbaecker KB, Vijai J*, et al.* Identification of a BRCA2-specific modifier locus at 6p24 related to breast cancer risk. PLoS Genet 2013;9(3):e1003173.

15. Kuchenbaecker KB, Neuhausen SL, Robson M*, et al.* Associations of common breast cancer susceptibility alleles with risk of breast cancer subtypes in BRCA1 and BRCA2 mutation carriers. Breast Cancer Res 2014;16(6):3416.

16. Antoniou AC, Wang X, Fredericksen ZS*, et al.* A locus on 19p13 modifies risk of breast cancer in BRCA1 mutation carriers and is associated with hormone receptor-negative breast cancer in the general population. Nat Genet 2010;42(10):885-92.

17. Barnes DR, Lee A, Investigators E*, et al.* Evaluation of association methods for analysing modifiers of disease risk in carriers of high-risk mutations. Genet Epidemiol 2012;36(3):274-91.

18. Antoniou AC, Goldgar DE, Andrieu N*, et al.* A weighted cohort approach for analysing factors modifying disease risks in carriers of high-risk susceptibility genes. Genet Epidemiol 2005;29(1):1-11.

19. Antoniou AC, Rookus M, Andrieu N*, et al.* Reproductive and hormonal factors, and ovarian cancer risk for BRCA1 and BRCA2 mutation carriers: results from the International BRCA1/2 Carrier Cohort Study. Cancer Epidemiol Biomarkers Prev 2009;18(2):601-10.

20. Antoniou A, Pharoah PD, Narod S*, et al.* Average risks of breast and ovarian cancer associated with BRCA1 or BRCA2 mutations detected in case Series unselected for family history: a combined analysis of 22 studies. Am J Hum Genet 2003;72(5):1117-30.

21. Antoniou AC, Cunningham AP, Peto J*, et al.* The BOADICEA model of genetic susceptibility to breast and ovarian cancers: updates and extensions. Br J Cancer 2008;98(8):1457-66.

22. Somers RH. A New Asymmetric Measure of Association for Ordinal Variables. American Sociological Review 1962;27(6):799-811.

23. Harrell FE, Califf RM, Pryor DB*, et al.* Evaluating the Yield of Medical Tests. Jama-Journal of the American Medical Association 1982;247(18):2543-2546.

24. Harrell FE, Jr., Lee KL, Mark DB. Multivariable prognostic models: issues in developing models, evaluating assumptions and adequacy, and measuring and reducing errors. Stat Med 1996;15(4):361-87.

25. Newson RB. Comparing the predictive powers of survival models using Harrell's C or Somers' D. Stata Journal 2010;10(3):339-358.

26. Newson R. Parameters behind "nonparametric" statistics: Kendall's tau, Somers' D and median differences. Stata Journal 2002;2(1):45-64.

27. Antoniou AC, Beesley J, McGuffog L*, et al.* Common breast cancer susceptibility alleles and the risk of breast cancer for BRCA1 and BRCA2 mutation carriers: implications for risk prediction. Cancer Res 2010;70(23):9742-54.

28. Amin Al Olama A, Benlloch S, Antoniou AC*, et al.* Risk Analysis of Prostate Cancer in PRACTICAL, a Multinational Consortium, Using 25 Known Prostate Cancer Susceptibility Loci. Cancer Epidemiol Biomarkers Prev 2015;24(7):1121-9.
